# Population connectivity buffers genetic diversity loss in a seabird

**DOI:** 10.1186/1742-9994-10-28

**Published:** 2013-05-20

**Authors:** Oscar Ramírez, Elena Gómez-Díaz, Iñigo Olalde, Juan Carlos Illera, Juan Carlos Rando, Jacob González-Solís, Carles Lalueza-Fox

**Affiliations:** 1 Institute of Evolutionary Biology (CSIC-Universitat Pompeu Fabra), Barcelona, Spain; 2 Research Unit of Biodiversity (UO-CSIC-PA), Oviedo University, Asturias, Spain; 3 Departamento de Biología Animal (UDI Zoología), Universidad de La Laguna, La Laguna, Tenerife, Spain; 4 Institut de Recerca de la Biodiversitat and Departament de Biologia Animal, Universitat de Barcelona, Barcelona, Spain

**Keywords:** Ancient DNA, Population size, *Calonectris*

## Abstract

**Background:**

Ancient DNA has revolutionized conservation genetic studies as it allows monitoring of the genetic variability of species through time and predicting the impact of ecosystems’ threats on future population dynamics and viability. Meanwhile, the consequences of anthropogenic activities and climate change to island faunas, particularly seabirds, remain largely unknown. In this study, we examined temporal changes in the genetic diversity of a threatened seabird, the Cory’s shearwater (*Calonectris borealis*).

**Findings:**

We analysed the mitochondrial DNA control region of ancient bone samples from the late-Holocene retrieved from the Canary archipelago (NE Atlantic) together with modern DNA sequences representative of the entire breeding range of the species. Our results show high levels of ancient genetic diversity in the Canaries comparable to that of the extant population. The temporal haplotype network further revealed rare but recurrent long-distance dispersal between ocean basins. The Bayesian demographic analyses reveal both regional and local population size expansion events, and this is in spite of the demographic decline experienced by the species over the last millennia.

**Conclusions:**

Our findings suggest that population connectivity of the species has acted as a buffer of genetic losses and illustrate the use of ancient DNA to uncover such cryptic genetic events.

## Findings

Over the last millennia island faunas have been progressively decimated by human exploitation, biological invasions and habitat destruction [1]. Deterministic threats of that kind have direct mortality effects on island populations leading to short-term demographic declines. This would in turn lead to a loss of genetic diversity and an increase of genetic stochasticity in small populations, such as inbreeding and population bottlenecks, ultimately increasing the probability of extinction [2]. This global process is particularly well exemplified among seabirds in general and albatrosses and petrels in particular, hereafter Procellariiforms [3]. During the Holocene at least 17 Procellariiform species have vanished [4], and 56% of the extant species are known or suspected to be experiencing population declines [5]. In the Canary Islands this process has led to the extinction of the dune shearwater (*Puffinus holeae*), the lava shearwater (*P. olsoni*), and an unidentified species of petrel (*Pterodroma* sp.) [6-8].

One of the most abundant Procellariform species inhabiting the Canary archipelago is the Atlantic Cory´s shearwater (*Calonectris borealis*). The breeding population of the species has been estimated as more than 270,000 pairs, however it underwent an acute decline between 1970–1990 and currently it continues to decline [9]. This species faces a number of conservation threats globally, both at sea (i.e. fisheries by-catch) and at land (i.e. animal invasions), which have been shown to seriously impact adult survival and reproductive success (see [10]). In the Canary Islands the Cory’s shearwaters have been by and large exploited as a food resource by the aboriginal peoples who reached the islands sometime between 756 cal BC - 313 cal AD and has also suffered predation by introduced rats and cats for centuries [11]. This has possibly led to a population bottleneck. Nevertheless, the impact of these threats on the genetic status of the species is unknown.

Ancient DNA (aDNA) has revolutionised biodiversity and conservation studies because it provides an excellent opportunity to monitor the genetic variability of species or populations through time [12]. However, few studies to date have used such heterochronous' data sets for conservation purposes in seabirds [13,14]. This is partly due to the inherent methodological difficulties of aDNA research [15], but also to the scarcity of fossil remains in remote oceanic archipelagos.

Using an heterochronous sampling consisting of Cory’s shearwater bone samples from late-Holocene from the Canary Islands together with current sequences representative of the entire breeding range of two sister *Calonectris* species, the Cory’s and the Scopoli’s (*C. diomedea*) shearwaters, we aim: (*i*) to estimate levels of genetic diversity and relationships between ancient and modern Cory’s shearwater populations, and (*ii*) to reconstruct the population demographic history of the Cory’s shearwater and estimate population size changes within the Canary archipelago through time.

**Figure 1:**
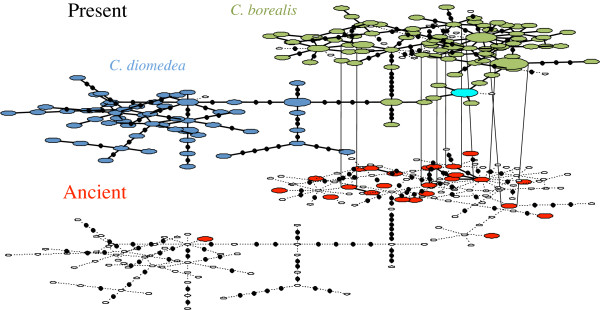
**Heterochronous haplotype network for ancient (in red) and modern *Calonectris *shearwaters (the only haplotype shared between the two sister taxa, Cory’s and Scopoli's shearwaters, is indicated in bright blue).** The five haplotypes shared between the two-epoch levels are connected. Small empty ellipses represent haplotypes from one level projected on the opposing haplotype network, then providing a visual representation of the genetic diversity change through time. Black dots represent mutational steps.

All collected bones were morphologically identified as belonging to the Cory’s shearwater species. ^14^C dates show that the three samples analyzed are from the last millennium. The 2σ confidence interval of two samples fall between 1004–1530 cal yr AD being the third younger than 1499 cal AD (Additional file 1: Table S1). DNA from pooled ancient samples (N=14) resulted in 25 mtDNA distinct haplotypes (Additional file 2: Figure S1). Only three subsamples contained one single haplotype, and the remaining subsamples presented two haplotypes each. The authenticity of these 25 haplotypes is supported by: 1) negative controls at the extraction and PCR stages were uniformly clean, 2) all the fragments have been replicated, by independent PCR, at least twice and on average 3.5 times (Additional file 2: Figure S1), 3) ancient sequences obtained clearly cluster within the extant Cory’s shearwater gene pool (Figure 1 and 4) all haplotypes were unique to single subsamples, indicating that there had been no cross contamination between samples/sites.

The genetic diversity values were high and similar between the ancient and the modern population (Table 1). Most individuals presented unique haplotypes and only five of the 25 haplotypes detected in the pooled ancient samples correspond to haplotypes still present in current populations, two of them being nested within the most common modern haplotypes (Figure 1). The remaining 20 ancient haplotypes were not present in any of the 282 extant Cory’s shearwaters analysed, including the 42 newly analysed individuals from Montaña Clara [16,17] , see Additional file 3: Table S2.

**Table 1:** Genetics statistics for the extant and extinct populations of Cory’s and Scopoli’s shearwaters

**Populations**	**Sample size**	**Haplotypes**	**Nucleotide diversity (SD)**	**Theta (θ)**
Total	185	161	0.05579 (0.00146)	0.05944
*C. diomedea*	65	60	0.04120 (0.00262)	0.05270
*C. borealis*	121	103	0.03556 (0.00192)	0.05484
*C. borealis* (All M. Clara-Lanzarote)	61	54	0.03368 (0.00240)	0.05461
*C. borealis* (M. Clara-Lanzarote extant)	37	32	0.02919 (0.00226)	0.04126
*C. borealis* (M. Clara-Lanzarote extinct)	≥ 25	25	0.03861 (0.00455)	0.05591

The temporal haplotype network obtained illustrates the high genetic diversity found in both ancient (Atlantic) and modern samples, and agrees with the marked genetic differentiation between the two *Calonectris* shearwaters sister clades, so all ancient samples clustered with Atlantic Cory’s shearwater extant haplotypes. However, there is one ancient haplotype that grouped within the Mediterranean Scopoli’s cluster (Figure 1). These findings are concordant with those found in a previous study on modern samples [16], that show various introgression and migration events as well as low but significant levels of interspecific gene flow [16,17]. Our results on the ancient samples add further evidence of the rare albeit recurrent hybridization events between the two species.

**Figure 2:**
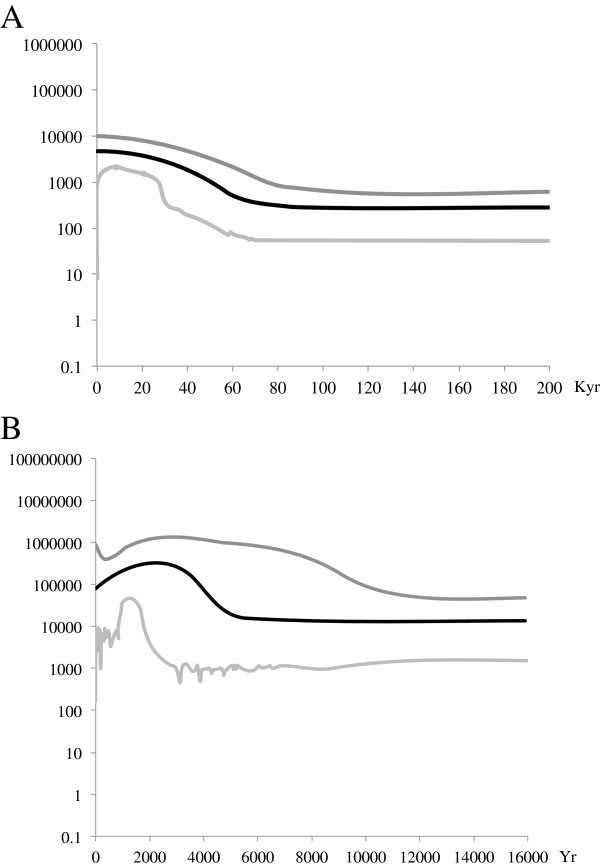
**Demographic history of the (A) Cory’s shearwater, and (B) Canary archipelago population.** EBSP showing changes in effective population size (Ne) through time and a piecewise-linear model. The y-axis representing the effective population size is given on a logarithmic scale. The thick solid black line is the median estimate, and area delimited by the upper and lower grey lines represents the HPD 95% confidence intervals for the effective population size.

The demographic analysis on the Cory’s shearwater indicated significant departures from a constant population model with the majority of credible sets containing non-zero population size changes. The Extended Bayesian Skyline Plot (EBSP) reconstruction of changes in genetic diversity through time shows a population expansion event at about 50,000 yr (Figure 2A). At a local scale and using radio-carbon rates to calibrate the demographic time-function (see Additional file 4: Supplementary methods), the EBSP analysis suggests a slight increase in genetic diversity in the Canary Archipelago over the last five millennia (Figure 2B).

Overall, these findings would not support the initial hypothesis of a loss in genetic variability in the Cory’s shearwater, in spite of its marked demographic population decline over the last millennium in the Canaries [10]. Several factors such as the high dispersal ability, the high effective population size, and the long generation time of the species may have contributed to offset the loss caused by these anthropogenic impacts [18]. The finding that high connectivity can buffer genetic diversity in the face of a demographic decline, has been reported in marine mammals (i.e. [19]), which have in common with seabirds the ability to travel long distances. This hypothesis is further supported by previous genetic and ecological data on the species that show high levels of population connectivity at both regional and local scales [16]. While the potential for dispersal will likely help the persistence of the colony in the short-term, future viability is debatable considering the life-history traits of this species. That is, *Calonectris* shearwaters share the typical and extreme life history traits of Procellariiform seabirds: long-lived, low fecundity and high survival, and thereby the species is particularly susceptible to threats increasing adult mortality [20]. In this regard, buffering effects on the genetic diversity may not be enough on a long-term basis if threats affecting the declining of populations persist. More generally, our study illustrates how aDNA can assist to better understand how populations function and evolve across time and so predict the impact of future anthropogenic and environmental changes on the seabird community, which is essential for implementing effective management strategies.

## Methods

**Figure 3:**
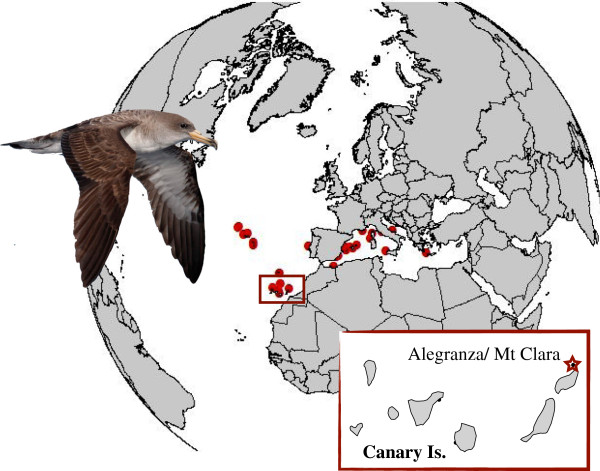
**Map of the *Calonectris *shearwaters sampling.**

All experimental procedures on ancient samples were performed in a dedicated aDNA laboratory (IBE-PRBB, Barcelona), where no previous work with extant *Calonectris* shearwaters had been conducted. Ancient bone fragments were collected in Montaña Clara and Alegranza islets in the northeastern part of the Canaries (Figure 3). All samples were collected from 10 to 50 cm from the surface and separated by sampling location. Three of the best-preserved bones from Montaña Clara were dated by accelerator mass spectrometer radiocarbon analysis (AMS ^14^C). The rest of the bones were subsequently used for genetic analysis. Due to the fragmentary nature of the remains, the bones of different specimens could not be individualized. Subsequently, we generated fourteen subsamples (one for each of the fourteen sampling places) of pooled skeletal remains of similar weight (around 150 mg) aiming to identify *a posteriori* the different mitochondrial (mtDNA) haplotypes present on each subsample analysed. This pooling strategy was imposed by the limitation in size of the remains, and although it does not allow determination of the precise number of individuals analysed (only a minimum of 25, see results), it is still a useful inexpensive way to estimate the genetic diversity within a population [21]. The fourteen pooled bones were then ground to powder and used for DNA extraction as described elsewhere [22]. We amplified a fragment of 218 bp of the mtDNA control region (see Additional file 5: Table S3). GenBank accessions numbers KC888879– KC888904.

The modern samples included 240 mtDNA control region sequences published in previous studies from across the entire distribution range of the two sister species, the Cory’s and the Scopoli’s shearwaters [16,17]. A subset of 42 modern samples from Montaña Clara (Lanzarote, Canary Is.) were further amplified and included in the analyses. For these samples, DNA extraction and amplification methods were performed at IBE-CMIMA laboratory (Barcelona) as described elsewhere [17]. See Additional file 3: Table S2 for further details on the dataset. GenBank accessions numbers KC888905–KC888924.

Genetic statistics for both modern and ancient samples were performed in the program DnaSP, v. 5.10.01 [23]. To depict the genealogical relationships between ancient and modern DNA samples and the continuity of haplotypes across time, a temporal network was constructed using TempNet [24]. For this analysis and to facilitate data interpretation, only unique haplotypes were considered.

To investigate the demographic history of Cory’s shearwaters from the Canary archipelago, we estimated the shape of the population growth function through time by constructing Extended Bayesian Skyline Plots (EBSP) as implemented in BEAST v1.7.4 [25] (Additional file 1: Table S1; Additional file 2: Figure S1; Additional file 3: Table S2; Additional file 4: Supplementary methods; Additional file 5: Table S3).

## Competing interests

The authors declare that they have no competing interests.

## Authors’ contributions

OR, EG-D, JG-S, JCI, JCR and CL-F contributed to the design of this research. OR, EG-D, and IO performed the experimental analyses. OR, EG-S and JCI performed the analysis. OR, EG-D, JCR, JCI, JG-S and CL-F wrote the manuscript. All authors read and approved the final manuscript.

## Supplementary Material

Additional file 1: Table S1 Conventional radiocarbon ages (yr BP) and 2σ calibration intervals (cal AD) from 3 bones of the Cory's Shearwater (*Calonectris borealis*) from Montaña Clara Islet (Lanzarote, Canary Islands). The lower value of each interval must be considered a maximum the age of each bone. Click here for file

Additional file 2: Figure S1 Alignment of a 218 bp fragment corresponding to the mtDNA control region obtained in the 14 pooled ancient samples of the Cory's shearwaters from the Canaries. Click here for file

Additional file 3: Table S2 *Calonectris* specimens from the extant and the extinct populations included in this study (307 in total)*.* The majority of the modern samples (240 out of 282); are part of a genetic study previously published by the authors [16]. The GenBank accession numbers, as well as the geographic origin for each individual sequence, are indicated. Click here for file

Additional file 4 Supplementary Methods. Click here for file

Additional file 5: Table S3 Primers used for the amplification of a fragment corresponding to the mtDNA control region. Click here for file
