# Supplementary material for: Population connectivity buffers genetic diversity loss in a seabird
Source: Front Zool. 2013 May 20;10:28. doi: 10.1186/1742-9994-10-28 (PMC3662614; doi:10.1186/1742-9994-10-28)
Supplement: Additional file 1: Table S1 — Conventional radiocarbon ages (yr BP) and 2σ calibration intervals (cal AD) from 3 bones of the Cory's Shearwater (Calonectris borealis) from Montaña Clara Islet (Lanzarote, Canary Islands). The lower value of each interval must be considered a maximum the age of each bone. [file 1742-9994-10-28-S1.docx]

Supplementary Methods

Radiometric dating

Samples were dated using accelerator mass spectrometry (AMS) radiocarbon dating. The AMS radiocarbon date given in years “BP” are conventional radiocarbon ages with standard error, where “BP” is before present, being present the year 1950, following standard reporting procedures.Radiocarbon calibration was calculated by using the software OxCal v4.1 (ORAU 2012), the marine 09.14C calibration curve and a reservoir correction of ΔR = 157 ± 116 derived from the available 20 closest marine samples to the origin of the dated samples (reservoir corrections for the world oceans can be found at the Marine Reservoir Correction Database <http://calib.qub.ac.uk/marine/>). Date coming from calibration is expressed as 2σ interval (95.4% confidence) and given as “cal AD” being “AD” *Annus Domini*.

Mitochondrial Sequencing

Primers (Table S2) were designed using the published sequences of extant *Calonectris* sp [S1,S2]. Amplified products were obtained using a two-step PCR protocol [S3]and purified with a gene clean silica method using the DNA Extraction Kit and cloned using the Topo TA Cloning Kit (Invitrogen, The Netherlands) and subsequently sequenced with an Applied BioSystems 3100 DNA sequencer (Universitat Pompeu Fabra, Barcelona).

Data Analysis

BEAST estimates demographic parameters under the assumption of a single population. In this study all samples (ancient and modern) were included in the analysis. Best-fit models of nucleotide substitution were inferred using jModelTest v0.1 [S4]. First, we used a relaxed uncorrelated lognormal molecular clock assuming a substitution rate of 0.21 for Domain I of the mitochondrial controlregion of birds [S5]. Second, ages of the genetic sequences were also approximated using radiocarbon dates estimated from the ancient samples using the leaf-dating method approach implemented in BEAST 1.7.4 [S6,S7]. For this analysis, we used a log-normal distribution representing the 95% confidence interval of the radiocarbon dates (between 1009 and 1499 yr, see table S1) as a prior on the sampling time for ancient samples. We performed four different replicate runs using the piecewise-linear model for 1x10^8^ generations, sampling every 2,000 generations. Convergence for all model parameters was assessed using Tracer v1.5 [S8] after obtaining an effective sample size (ESS) > 200.

REFERENCES

S1 Gómez-Díaz, E., González-Solís, J. &Peinado, M.A. 2009 Population structure in a highly pelagic seabird, the Cory’s shearwater Calonectrisdiomedea: anexamination of genetics, morphology and ecology. *Mar. Ecol.-Prog. Ser.***382**, 197–209.

S2 Gómez-Díaz, E., González-Solís, J., Peinado, M.A. & Page, R.D.M. 2006 Phylogeographyof the Calonectris shearwaters using molecular and morphometric data.*Mol. Phylogenet. Evol.***41**, 322-332.

S3 Krause, J., Dear, P.H., Pollack, J.L., Slatkin, M., Spriggs, H., et al. 2006 Multiplex

amplification of the mammoth mitochondrial genome and the evolution ofElephantidae*Nature***439**, 724–727.

S4 Posada, D. 2008 jModelTest: Phylogenetic model averaging. *Mol BiolEvol***25**, 1253

S5 Wenink, P.W., Baker, A.J., &Tilanus, M.G. 1993Hypervariable-control-region sequences reveal global population structuring in a long-distance migrant shorebird, the Dunlin (*Calidris alpina*) *PNAS* **90**, 94-98

S6 Ho, S.Y.W. & Shapiro, B. 2011 Skyline-plot methods for estimating demographic history from nucleotide sequences.*Mol Ecol Res***11**, 423

S7 Shapiro, B., Ho, S.Y.W., Drummond, A.J., Suchard, M.A., Pybus, O.G., &Rambaut, A. 2011 A Bayesian Phylogenetic Method to Estimate Unknown Sequence Ages. Mol. Biol.Evol.**28**, 879-887

S8 Rambaut A. & Drummond A.J. 2007 Tracer v1.4, Available from http://beast.bio.ed.ac.uk/Tracer
