# Supplementary material for: Population connectivity buffers genetic diversity loss in a seabird
Source: Front Zool. 2013 May 20;10:28. doi: 10.1186/1742-9994-10-28 (PMC3662614; doi:10.1186/1742-9994-10-28)
Supplement: Additional file 2: Figure S1 — Alignment of a 218 bp fragment corresponding to the mtDNA control region obtained in the 14 pooled ancient samples of the Cory's shearwaters from the Canaries. [file 1742-9994-10-28-S2.docx]

**Figure S1.** Alignment of a 218 bp fragment corresponding to the mtDNA control region obtained in the 14 pooled ancient samples of *C. borealis*

**Pool: Alegranza1**

>FJ755610H128 CAAACACATTCAATGCATGGACACAATACATACCTCCCACGCGGATTATCTCTCTCCACTCCCCGGCCGGAACACAAGCACCCTTAAGCCCAATAGTCCCTAGTACCAGAAACTATCTGTCCTCGTACTGAAACCTACCTAACTCTTTCTCTGCACACCCCAAATCTTCCTAGATACGGATATACTCAACCACCTAAAATCAATCGTAGCAGGACAAA

>Alegranza1_8 .................T............................................................................C..................................T.........T.T......................................

>Alegranza1_7 ...G.............T............................................................................C..............C...................T..................................................

>Alegranza1_5 ...G.............T.....................T.T..T..................................TT............................C....T..............T..........T.....T............................C....

>Alegranza1_4 ...G.............T............................................................................C..................................T..................................................

>Alegranza1_1 ...............................................................T.............................................C...................T..................................................

>Alegranza1_3 ...............................................................T......................................T....................T.....T..................................................

>Alegranza1_2 ...G...........................................................T.............................................C...................T..................................................

>Alegranza1_7 CAAACACATTCAATGCATG...G.............T.............................................T..............................C..................................T.........T.T...C.............................C....TCAATCGTAGCAGGACAAA

>Alegranza1_2 ...............................................................T.............................................C...................T..........T......C...........................C....

>Alegranza1_3 ...G.............T............................................................................C..................................T.........T.T...C.............................C....

>Alegranza1_6 ......................................G.......................................................C..................................T.........T.T...C.............................C....

>Alegranza1_5 ...G.............T............................................................................C..................................T.........T.T...C.............................C....

>Alegranza1_1 ...G.............T...............................................................................T...........C...................T............T....C...........................C....

>Alegranza1_4 ...............................................................T.............................................C...................T..........T......C...........................C....

>Alegranza1_B07CAAACACATTCAATGCATGA........................................................C..................GTCCCTAGTACCAGAAAC

>Alegranza1_F08 .................T.....................................T....................

>Alegranza1_C08 .................T.......................................C..................

>Alegranza1_G08 ...G...........................................................T............

>Alegranza1_G07 .............................................................T.T............

>Alegranza1_H07 ..................T....................................T.....T.T............

>Alegranza1_H08 ...G.............T..........................................................

>Alegranza1_B08 ..........................................................A....T............

>Alegranza1_59 CAAACACATTCAATGCATG...G.............T.........................T...................T............GTCCCTAGTACCAGAAAC

>Alegranza1_43 ....................................................G..........T............

>Alegranza1_60 ...............................................................T............

>Alegranza1_47 ...G.............T..........................................................

>Alegranza1_34 ...............................................................T............

>Alegranza1_42 .................T.............................................T............

>Alegranza1_37 ...G.............T...A......................................................

>Alegranza1_44 ...........................................T...................T............

>Alegranza1_46 ....................................................G.......................

>Alegranza1_36 .................T.............................................T............

>Alegranza1_51 ......................T........................................T............

>Alegranza1_45 ..................T..................................T.........T............

>Alegranza1_54 .................T.............................................T............

>Alegranza1_39 ....................................................G..........T............

>Alegranza1_35 ...G................................TT....T........GG.......................

>Alegranza1_49 ...G.............T..........................................................

>Alegranza1_57 .........................................TT....T.......T.......T............

>Alegranza1_50 .................T......................................G......T............

>Alegranza1_55 ........................A......................................T............

>Alegranza1_41 ..T.............T..............................................T............

>Alegranza1_58 .........................................T.....................T............

>Alegranza1_53 ...............G.................................AA............T............

>Alegranza1'24 CCCTTAAGCCCAATAGTCC.............................C...................T..........T......C...........................C....TCAATCGTAGCAGGACAAA

>Alegranza1'18 .............................C...................T..........T..................................C....

>Alegranza1'26 ..............C..................................T.........T.T...C..........................G..C....

>Alegranza1'22 ..............C.........N........................T..........T......C........................N..C....

>Alegranza1'19 .............................C...................T..........T......N..N........................C....

>Alegranza1'23 .............................C...................T.........T.T...C.............................C....

>Alegranza1'21 ..............C..................................T.........T.T...C..............G..............C....

>Alegranza1'25 ..............C.........N........................T.........T.T...C.............................C....

>Alegranza1'20 .............................C....N..............T..........T......C...........................C....

>Alegranza1_C05 CCCTTAAGCCCAATAGTCC..............C..................................T..............................T.T............C....TCAATCGTAGCAGGACAAA

>Alegranza1_F05 .............................C...................T..........T..................................C....

>Alegranza1_B05 .............................C...................T..........T..................................C....

>Alegranza1_G05 ..............C..................................T..............................T.T............C....

>Alegr1Cons1 GACGCAATACATACCTCTCACGCGGATTATCTCTCTCCACTCCCCGGCCGGAACACAAGCACCCTTAAGCCCAATAGTCCCTAGTACCAGAAACCATCTGTCCTCGTACTGAAACCTACCTAACTCTTTTTCTGCACACTCTAAACCTTCCTAGATACGGATATACTCAACCACCCAAAA

>Alegr1Cons2 GACACAATACATACCTCCCACGCGGATTATCTCTCTCCACTCCCCGGCCGGAACACAAGCACCTTTAAGCCCAATAGTCCCTAGTACCAGAAACTATCTGTCCTCGTACCGAAACCTACCTAACTCTTTTTCTGCACACCTCAAATCCTCCTAGATACGGATATACTCAACCACCCAAAA

**Pool: Alegranza2**

>FJ755611Hap129 CAAACACATTCAATGCATGGACACAATACATACCTCTCACGCGGATTATCTCTCTCCACTCCCCGGCCGGAACACAAGCACCCTTAAGCCCAATAGTCCCTAGTACCAGAAACCATCTGTCCTCGTGCTGAAACCTACCTAACTCTTTTTCTGTACACCCTAAACCTTCCTAGATACGGATATACTCAACCACCCAAAATCAATCGTAGCAGGACAAA

>Alegranza2_64 ..............................................................................................T............A.CA..................C....C.............................................

>Alegranza2_63 ..............................................................................................T............A.....................C....C.....TC...T.C................................

>Alegranza2_57 ...............................................T..............................................T..T.........A.C...................C....C.............................................

>Alegranza2_60 ..............................................................................................T............A.....................C....C.....TC...T.C................................

>Alegranza2_58 ................T.............................................................................T............A.....................C....C.....TC...T.C................................

>Alegranza2_61 ..............................................................................................T............A.C...................C....C.............................................

>Alegranza2_59 ................T..............................T..............................................T............A.....................C....C.....TC...T.C................................

>Alegranza2_62 ................T.............................................................................T............A.C...................C....C.............................................

>Alegranza2_13 CAAACACATTCAATGCATG............................................................................GTCCCTAGTACCAGAAAC

>Alegranza2_22 ............................................................................

>Alegranza2_14 ............................................................................

>Alegranza2_19 ............................................................................

>Alegranza2_29 ............................................................................

>Alegranza2_09 ............................................................................

>Alegranza2_06 ............................................................................

>Alegranza2_15 ............................................................................

>Alegranza2_27 ............................................................................

>Alegranza2_18 ............................................................................

>Alegranza2_25 ....T.......................................................................

>Alegranza2_17 ............................................T...............................

>Alegranza2_21 .........T.....T.......................................................T....

>Alegranza2_20 .G..........................................................................

>Alegranza2_26 ............................................T...............................

>Alegranza2_10 ..........C.................................................................

>Alegranza2_35 CCCTTAAGCCCAATAGTCC..............T............A.C...................C....C.............................................TCAATCGTAGCAGGACAAA

>Alegranza2_34 ..............T............A.....................C....C..........T.C................................

>Alegranza2_36 ..............T............A.C...................C....C.............................................

>Alegranza2_C04 CCCTTAAGCCCAATAGTCC..............T............A.....................C....C.....TC...T.C................................TCAATCGTAGCAGGACAAA

>Alegranza2_D04 ..............T............A.C...................C....C.............................................

>Alegranza2_G04 ..............T............A.C...................C....C.............................................

>Alegranza2_H04 ..............T............A.C...................C....C.............................................

>Alegranza2_B04 ..............T............A.C...................C....C.............................................

>Alegr2Cons1 GACACAATACATACCTCTCACGCGGATTATCTCTCTCCACTCCCCGGCCGGAACACAAGCACCCTTAAGCCCAATAGTCCCTAGTACCAGAAACTATCTGTCCTCGTACCGAAACCTACCTAACTCTTTCTCTGCACACCCTAAACCTTCCTAGATACGGATATACTCAACCACCCAAAA

>Alegr2Cons2 GACACAATACATACCTCTCACGCGGATTATCTCTCTCCACTCCCCGGCCGGAACACAAGCACCCTTAAGCCCAATAGTCCCTAGTACCAGAAACTATCTGTCCTCGTACTGAAACCTACCTAACTCTTTCTCTGCACACCTCAAATCCTCCTAGATACGGATATACTCAACCACCCAAAA

**Pool: Alegranza3**

>FJ755592H110 CAAACACATTCAATGCATGGACACAACACATACCTCTCACGCGGATTATCTCTCTCCACTCCCCGGCCGGAACACAAGCACCCTTAAGCCCAATAGTCCCTAGTACCAGAAACTATCTGTCCTCGTACTGAAACCTACCTAACTCTTTCTCTGTACACCCCAAACCTTCCTAGATACGGATATACTCAACCACCTAAAATCAATCGTAGCAGGACAAA

>Alegranza3_14 ................T.T.......................T.T................T.......T..................................T........................................TTC.......G..NNNNNNN..........C....

>Alegranza3_12 ....................................T..................T...T...T............................................T.........T.....................T....T.C...........................C....

>Alegranza3_16 ............................................................................................................................................T....T.C...........NNNNNN..........C....

>Alegranza3_9 ......................................................................................................................................C.........................NNN............C....

>Alegranza3_11 ............................................................................................................................................TT...T.C..T........NNNNNN..........C....

>Alegranza3_15 ................T.................T...................................................................................................C.......................NNNNNNN..........C....

>Alegranza3_10 ..T..........................................................................................T..............................................T....T.C..........NNNNNN...........C....

>Alegranza3_13 ............................................T..................................T.T....................................................C........................NNNNN...........C....

>Alegranza3_14 CAAACACATTCAATGCATG.......T.......................C.....T........................................................C.......................................C.....T....T.C..................C.C......C....TCAATCGTAGCAGGACAAA

>Alegranza3_10 ....................................................................A.......................................................................T....T.C...........................C....

>Alegranza3_16 ......................................................................................................................................C........................................C....

>Alegranza3_11 ......................................................................................................................................C........................................C....

>Alegranza3_12 ....................T.T..............T...T.TT...TGG..........................................TT.............................................T....T.C...........................C....

>Alegranza3_15 ......................................................................................................................................C......................................T.C....

>Alegranza3_9 ...................................................................................................................T........................T....T.C...........................C....

>Alegranza3_13 ............................................................................................................................................T....T.C...........................C....

>Alegranza3_52 CAAACACATTCAATGCATG............................................................................GTCCCTAGTACCAGAAAC

>Alegranza3_56 ............................................................................

>Alegranza3_64 ....................................................T.......................

>Alegranza3_61 ..........................................................A.................

>Alegranza3_50 ......................T...........T..................T......................

>Alegranza3_62 ...........................................T................................

>Alegranza3_58 ............................................T..........T....................

>Alegranza3_57 ....................................................................A.......

>Alegranza3_54 .......................A....................................................

>Alegranza3_51 ......................T...........T..................T......................

>Alegranza3_60 .............T.........................................T.....T..............

>Alegranza3_53 ............................................................................

>Alegranza3_55 ............................................................................

>Alegranza3_49 ....................................................................A.......

>Alegranza3_59 ......................T............T........................................

>Alegr3Cons1 GACACAACACATACCTCTCACGCGGATTATCTCTCTCCACTCCCCGGCCGGAACACAAGCACCCTTAAGCCCAATAGTCCCTAGTACCAGAAACTATCTGTCCTCGTACTGAAACCTACCTAACTCTTTCTCTGCACACCCCAAACCTTCCTAGATACGGATATACTCAACCACCCAAAA

>Alegr3Cons2 GACACAACACATACCTCTCACGCGGATTATCTCTCTCCACTCCCCGGCCGGAACACAAGCACCCTTAAGCCCAATAGTCCCTAGTACCAGAAACTATCTGTCCTCGTACTGAAACCTACCTAACTCTTTCTCTGTACACCTCAAATCCTCCTAGATACGGATATACTCAACCACCCAAAA

**Pool: Montaña1**

>FJ755565H83 CAAACACATTCAATGCATGGACACAATACATACCTCTCACGCGGATTATCTCTCTCCACTCCCCGGCCGGAACACAGGCACCCTTAAGCCCAATAGTCCCTAGTACCAGAAACTATCTGTCCTCGTACTGAAACCTACCTAACTCTTTCTCTGCACACCCTAAACCTTCCTAGATACGGATATACTCAACCACCCAAAATCAATCGTAGCAGGACAAA

>Montaña1_G11 ................T.................T.................................................................................................................................................

>Montaña1_C10 ................T.............T.T.T.T...............................................................................................................................................

>Montaña1_A10 ...............................................................T....................................................................................................................

>Montaña1_G10 ....................................................................................................................................................................................

>Montaña1_B12 ....................................................................................................................................................................................

>Montaña1_E10 ....................................................................................................................................................................................

>Montaña1_D10 .........................................................................................A..........................................................................................

>Montaña1_A11 ....................................................................................................................................................................................

>Montaña1_D11 ....................................................................................................................................................................................

>Montaña1_F12 ....................................................................................................................................................................................

>Montaña1_E11 ......................................................................................................T.............................................................................

>Montaña1_H10 ....................................................................................................................................................................................

>Montaña1_C12 ....................................................................................................................................................................................

>Montaña1_C11 ..........................................................C.........................................................................................................................

>Montaña1_B10 ....................................................................................................................................................................................

>Montaña1_F10 ................T.................T.................................................................................................................................................

>Montaña1_H11 ................T.................T.................................................................................................................................................

>Montaña1_D12 .............T...................................................................................T..................................................................................

>Montaña1_H12 .............T..T................................................................................T..................................................................................

>Montaña1_F11 ....................................................................................................................................................................................

>Montaña1_54 CAAACACATTCAATGCATG................T.................T.................................................................................................................................................TCAATCGTAGCAGGACAAA

>Montaña1_49 ..................................................................................................C.................................................................................

>Montaña1_50 ....................................................................................................................................................................................

>Montaña1_51 ....................................................................................................................................................................................

>Montaña1_53 ....................................................................................................................................................................................

>Montaña1_83 CAAACACATTCAATGCATG............................................................................GTCCCTAGTACCAGAAAC

>Montaña1_84 ............................................................................

>Montaña1_85 ............................................................................

>Montaña1_86 ............................................................................

>Montaña1_87 ............................................................................

>Montaña1_88 ............................................................................

>Montaña1_89 .............................................................T..............

>Montaña1_90 ............................................................................

>Montaña1_91 ............................................................................

>Montaña1_92 ............................................................................

>Montaña1_93 ............................................................................

>Montaña1_94 ............................................................................

>Montaña1_95 ............................................................................

>Montaña1_96 ............................................................................

>Montaña1_E03CAAACACATTCAATGCATG............................................................................GTCCCTAGTACCAGAAAC

>Montaña1_D04 ....................................................G.......................

>Montaña1_G03 ............................................................................

>Montaña1_E04 ............................................................................

>Montaña1_C04 ............................................................................

>Montaña1_G04 ............................................................................

>Montaña1_B04 ............................................................................

>Montaña1_B03 ............................................................................

>Montaña1_A03 ............................................................................

>Montaña1_H04 ....................................................G.......................

>Montaña1_F04 ............................................................................

>Montaña1_D03 ..............T..C..........................................................

>Montaña1_F03 ............................................................................

>Montaña1_C03 ............................................................................

>Montaña1 CCCTTAAGCCCAATAGTCC......................................................................N........................N....TCAATCGTAGCAGGACAAA

>Montaña1'30 ....................................................................................................

>Montaña1'31 ....................................................................................................

>Montaña1'29 ....................................................................................................

>Montaña1'34 ....................................................................................................

>Montaña1'39 ....................................................................................................

>Montaña1'32 ........................N.............................................N.........N.NN.N...N..N.......

>Montaña1'36 ......................................................................N.............................

>Montaña1'33 ........................N........................................................N..................

>Montaña1'38 ......................................................................N.............................

>Montaña1_E01 CCCTTAAGCCCAATAGTCC....................................................................................................TCAATCGTAGCAGGACAAA

>Montaña1_F01 ....................................................................................................

>Montaña1_E02 ....................................................................................................

>Montaña1_B01 ....................................................................................................

>Montaña1_F02 ....................................................................................................

>Montaña1_C02 ....................................................................................................

>Montaña1_H01 ....G...............................................................................................

>Montaña1_B02 ....................................................................................................

>Montaña1_A01 ...............................................................................C....................

>Montaña1_H02 ....................................................................................................

>Montaña1_A02 T...................................................................................................

>Montaña1_G01 ....................................................................................................

>MC1Cons GACACAATACATACCTCTCACGCGGATTATCTCTCTCCACTCCCCGGCCGGAACACAGGCACCCTTAAGCCCAATAGTCCCTAGTACCAGAAACTATCTGTCCTCGTACTGAAACCTACCTAACTCTTTCTCTGCACACCCTAAACCTTCCTAGATACGGATATACTCAACCACCCAAAA

**Pool: Montaña1.2**

FJ755506.1H24 CAAACACATTCAATGCATGAATCCAATACATACCTCTCACGCGGATTATCTCTCTCTACTCCCCGGTCGGAACACAAGCACCCTTAAGCCCAATAGTCCCTAGTACCAGAAACCATCTGCCCTCGTGCCTAAACCTACCTAACTCTTTCTCTGTACACTCCAAATCCTCCTAGATACGGATATACTCCACCACCCAAAATCAATCGTAGCAGGACAAA

>Montaña1.2_7 G.CA............T.................T..C.........C..............................................T.....T......A.TG.......................C....CT.........................C.A...........

>Montaña1.2_8 G.CA.................................C.........C..............................................T.....T......A.TG.......................C....CT.........................C.A...........

>Montaña1.2_1 ....................................T.................................................................................................................................C.............

>Montaña1.2_3 ....................................T...............................................................................................................................................

>Montaña1.2_16 G.CA.................................C.........C..............................................T.....T......A.TG.......................C....CT.........................C.A...........

>Montaña1.2_6 G.CA..............................T..C.........C.........G....................................T.....T......A.TG.......................C....C.T...C.T....................A...........

>Montaña1.2_11 G.CA.................................C.........C..............................................T.....T......A.TG.......................C....CT.........................C.A...........

>Montaña1.2_12 G.CA.................................C.........C..............................................T.....T......A.TG.......................C....CT.........................C.A...........

>Montaña1.2_10 ....................................T...............................................................................................................................................

>Montaña1.2_13 ...T................................T...............................................................................................................................................

>Montaña1.2_9 G.CA............T.................T..C.........C..............................................T.....T......A.TG.......................C....CT.........................C.A...........

>Montaña1.2_5 G.CA.................................C.........C..............................................T.....T......A.TG.......................C....CT.........................C.A...........

>Montaña1.2_2 ....................................T...............................................................................................................................................

>Montaña1.2_15 G.CA............T.................T..C.........C..............................................T.....T......A.TG.......................C....CT.........................C.A...........

>Montaña1.2_4 G.CA............T.................T..C.........C..............................................T.....T......A.TG.......................C....CT.........................C.A...........

>Montaña1.2_14 ....................................T...............................................................................................................................................

>FJ755506.1H24 CAAACACATTCAATGCATGAATCCAATACATACCTCTCACGCGGATTATCTCTCTCTACTCCCCGGTCGGAACACAAGCACCCTTAAGCCCAATAGTCCCTAGTACCAGAAACCATCTGCCCTCGTGCCTAAACCTACCTAACTCTTTCTCTGTACACTCCAAATCCTCCTAGATACGGATATACTCCACCACCCAAAATCAATCGTAGCAGGACAAA

>Montaña1.2_D07 ....................................................................................................................................................................................

>Montaña1.2_G08 ....................................T...............................................................................................................................................

>Montaña1.2_H08 ....................................T...............................................................................................................................................

>Montaña1.2_A07 ....................................T...............................................................................................................................................

>Montaña1.2_D09 ....................................................................................................................................................................................

>Montaña1.2_B09 ....................................................................................................................................................................................

>Montaña1.2_E09 ....................................T...............................................................................................................................................

>Montaña1.2_C07 ....................................T...............................................................................................................................................

>Montaña1.2_A09 ....................................T...............................................................................................................................................

>Montaña1.2_B08 G.CA...........C....................T...............................................................................................................................................

>Montaña1.2_C09 ....................................T............A..................................................................................................................................

>Montaña1.2_B07 ....................................T...............................................................................................................................................

>Montaña1.2_F08 ....................................................................................................................................................................................

>Montaña1.2_D08 G.CA...........C.....................C.......A.C..............................................T.....T......A.TG...........G...........C....C.....C.T....................A...........

>Montaña1.2_C08 ....................................T...............................................................................................................................................

>Montaña1.2_H07 ....................................T...............................................................................................................................................

>Montaña1.2_G07 ....................................T...............................................................................................................................................

>Montaña1.2_G09 ....................................T...............................................................................................................................................

>Montaña1.2_A08 G.CA...........C.....................C.......A.C..............................................T.....T......A.TG...........G...........C....C.....C.T....................A...........

>Montaña1.2_F07 ....................................T...............................................................................................................................................

>Montaña1.2_E07 G.C............C.....................C.........C..............................................T.....T......A.TG...........G...........C....C.....C.T....................A...........

>Montaña1.2_F09 ....................................T...............................................................................................................................................

>FJ755506.1H24 CAAACACATTCAATGCATGAATCCAATACATACCTCTCACGCGGATTATCTCTCTCTACTCCCCGGTCGGAACACAAGCACCCTTAAGCCCAATAGTCCCTAGTACCAGAAACCATCTGCCCTCGTGCCTAAACCTACCTAACTCTTTCTCTGTACACTCCAAATCCTCCTAGATACGGATATACTCCACCACCCAAAATCAATCGTAGCAGGACAAA

>Montaña1.2_17 ....................................T...............................................................................................................................................

>Montaña1.2_21 ....................................T...............................................................................................................................................

>Montaña1.2_22 ....................................T...............................................................................................................................................

>Montaña1.2_23 ....................................T...............................................................................................................................................

>Montaña1.2_24 ....................................T...............................................................................................................................................

>Montaña1.2_25 ....................................T...............................................................................................................................................

>Montaña1.2_27 ....................................T...........................................................................................................................G...................

>Montaña1.2_28 ....................................T...............................................................................................................................................

>Montaña1.2_29 ....................................T...............................................................................................................................................

>Montaña1.2_30 ....................................T...............................................................................................................................................

>Montaña1.2_31 ....................................T...............................................................................................................................................

>Montaña1.2_32 ....................................................................................................................................................................................

>Montaña1.2_34 ....................................T...............................................................................C...............................................................

>Montaña1.2_35 .............................C......................................................................................................................................................

>Montaña1.2_36 ....................................T....................G..........................................................................................................................

>Montaña1.2_37 ....................................T...............................................................................................................................................

>Montaña1.2_42 ....................................................................................................................................................................................

>Montaña1.2_44 ..........................A.........................................................................................................................................................

>Montaña1.2_47 ....................................................................................................................................................................................

>Montaña1.2_48 ....................................................................................................................................................................................

>FJ755506.1H24 CCCTTAAGCCCAATAGTCCCTAGTACCAGAAACCATCTGCCCTCGTGCCTAAACCTACCTAACTCTTTCTCTGTACACTCCAAATCCTCCTAGATACGGATATACTCCACCACCCAAAATCAATCGTAGCAGGACAAA

>Montaña1.2_6 ....................................................................................................

>Montaña1.2_7 ....................................................................................................

>Montaña1.2_8 ....................................................................................................

>Montaña1.2_10 ....................................................................................................

MC1.2Cons1 AATCCAATACATACCTCTCACGCGGATTATCTCTCTTTACTCCCCGGTCGGAACACAAGCACCCTTAAGCCCAATAGTCCCTAGTACCAGAAACCATCTGCCCTCGTGCCTAAACCTACCTAACTCTTTCTCTGTACACTCCAAATCCTCCTAGATACGGATATACTCCACCACCCAAAA

MC1.2Cons2 GACACAATACATACCTCTCACGCGGATTATCTCTCTCCACTCCCCGGCCGGAACACAAGCACCCTTAAGCCCAATAGTCCCTAGTACCAGAAACTATCTGTCCTCGTACTGAAACCTACCTAACTCTTTCTCTGCACACCCCAAATCCTCCTAGATACGGATATACCCAACCACCCAAAA

**Pool: Montaña3.2**

>FJ755583H101 CAAACACATTCAATGCATGGACACAATACATACCCCTCACGCGGATTATCTCTCTCCACTCCCCGGCCGGAACACAAGCACCCTTAAGCCCAATAGTCCCTAGTACCAGAAACTATCTGTCCTCGTACTGAAACCTACCTAGCTCTTTCTCTGCACACCCCAAACCTTCCTAGATACGGATATACTCAACCACCCAAAATCAATCGTAGCAGGACAAA

>Montaña3.2_32 ....................T.................................................................................................T.............................................................

>Montaña3.2_23 .............................................................................................................................................................T......................

>Montaña3.2_18 ..................................T..................T..............................................................................................................................

>Montaña3.2_19 ..................................T..........A...........................................A..........................................................................................

>Montaña3.2_26 .............................................A...........................................A.......................................T..................................................

>Montaña3.2_25 ....................................................................................................................................................................................

>Montaña3.2_27 ...................................................................................A................................................................................................

>Montaña3.2_28 .............................................A.............T.......................................................................T................................................

>Montaña3.2_20 .............................................A........................................................................................T.............................................

>Montaña3.2_21 .........................................................................................A..........................................................................................

>Montaña3.2_29 .........................................TT..............................................................................................T..........................................

>Montaña3.2_17 ..................................T..........A...........................................A..........................................................................................

>Montaña3.2_22 .......................................................................T............................................................................................................

>Montaña3.2_31 ..................................T..........A...........................................A..........................................................................................

>FJ755583H101 CAAACACATTCAATGCATGGACACAATACATACCCCTCACGCGGATTATCTCTCTCCACTCCCCGGCCGGAACACAAGCACCCTTAAGCCCAATAGTCCCTAGTACCAGAAACTATCTGTCCTCGTACTGAAACCTACCTAGCTCTTTCTCTGCACACCCCAAACCTTCCTAGATACGGATATACTCAACCACCCAAAATCAATCGTAGCAGGACAAA

>Montaña3.2_H01 ....................T...............................................................................................................................................................

>Montaña3.2_G02 .............................................A...........................................A.......................................................T..................................

>Montaña3.2_E01 .............................................A...........................................A..........................................................................................

>Montaña3.2_C02 .......................................................................................................C..........................................T.................................

>Montaña3.2_B03 ..................................T.................................................................................................................................................

>Montaña3.2_D03 .............................................A...........................................A..........................................................................................

>Montaña3.2_D02 ....................................................................................................................................................................................

>Montaña3.2_E03 .............................................A...........................................A..........................................................................................

>Montaña3.2_D01 ..................................T..........A...................................................................................................T..................................

>Montaña3.2_A01 ..................................T..........A...........................................A..........................................................................................

>Montaña3.2_F03 ..........................................................A.........................................................................................................................

>Montaña3.2_B01 ....................................................................................................................................................................................

>Montaña3.2_G03 ......................T.............................................................................................................................................................

>Montaña3.2_H02 .............................................A...........................................A..............................................T...........................................

>Montaña3.2_C03 ..................................T..........A.....................................A.....A..........................................................................................

>Montaña3.2_A03 ...........................................T.A...........................................A.............................T............................................................

>Montaña3.2_C01 ...............T....................TT...........A........................................................................A.........................................................

>Montaña3.2_F02 ..................................T..........A...........................................A..........................................................................................

>Montaña3.2_H03 .............................................A..T........................................A..........................................................................................

>Montaña3.2_E02 ..................................T..........A...........................................A..........................................................................................

>Montaña3.2_F01 .............................................A...........................................A..........................................................................................

>FJ755583H101 CAAACACATTCAATGCATGGACACAATACATACCCCTCACGCGGATTATCTCTCTCCACTCCCCGGCCGGAACACAAGCACCCTTAAGCCCAATAGTCCCTAGTACCAGAAACTATCTGTCCTCGTACTGAAACCTACCTAGCTCTTTCTCTGCACACCCCAAACCTTCCTAGATACGGATATACTCAACCACCCAAAATCAATCGTAGCAGGACAAA

>Montaña3.2_75 ....................................................................................................................................................................................

>Montaña3.2_78 ..................................T..................T..........................................................................................................G...................

>Montaña3.2_67 .............................................A...........................................A..........................................................................................

>Montaña3.2_57 ..................................T..........A...........................................A..........................................................................................

>Montaña3.2_73 .................................C..............................................................C............................................................T......................

>Montaña3.2_64 ..................................T..........A...........................................A..........................................................................................

>Montaña3.2_52 ...........................................T..........................T.............................................................................................................

>Montaña3.2_95 ..................................T..........A...........................................A..........................................................................................

>Montaña3.2_55 ..................................T..........A...........................................A..........................................................................................

>Montaña3.2_88 ...........C........................................................................................................................................................................

>Montaña3.2_68 ..................................T..........A...........................................A..........................................................................................

>Montaña3.2_77 .............................................A...........................................A..........................................................................................

>Montaña3.2_86 .............................................................T......................................................................................................................

>Montaña3.2_92 ...................................................................................................A................................................................................

>Montaña3.2_63 ....................................................................................................................................................................................

>Montaña3.2_74 ....................................................................................................................................................................................

>Montaña3.2_58 ........................................................................................................................................T...........................................

>Montaña3.2_82 ..................................T..........A......................................................................................................................................

>Montaña3.2_49 .............................................A...........................................A..........................................................................................

>Montaña3.2_62 ....................................................................................................................................................................................

>Montaña3.2_71 ....................................................................................................................................................................................

>Montaña3.2_53 ............................................T...........................................................................................T...........................................

>Montaña3.2_72 ..................................T..........A...........................................A..........................................................................................

>Montaña3.2_80 ..................................T..........................................................T.................................................................T....................

>Montaña3.2_84 .............................................................T......................................................................................................................

>Montaña3.2_83 .............................................A...........................................A..........................................................................................

>Montaña3.2_51 ....................................................................................................................................................................................

>Montaña3.2_91 ....................................................................................................................................................................................

>Montaña3.2_66 .............................................A...........................................A..........................................................................................

>Montaña3.2_90 ......................................................................................................................T.............................................................

>Montaña3.2_61 ....................................................................................................................................................................................

>Montaña3.2_81 ..................................T..........A...........................................A...................................................................................T......

>Montaña3.2_60 .............................................A...........................................A..........................................................................................

>Montaña3.2_69 ..................................T.................................................................................................................................................

>Montaña3.2_94 ...............................................................................................................................................................A....................

>Montaña3.2_50 ..................................T..........................................................................................................................A......................

>Montaña3.2_59 .............................................A...........................................A..........................................................................................

>Montaña3.2_85 ....................................................................................................................................................................................

>Montaña3.2_87 .............................................A...........................................A..........................................................................................

>Montaña3.2_54 ..........................................................................................................................................T.........................................

>Montaña3.2_93 .............................C...............A...........................................A..........................................................................................

>Montaña3.2_70 ..................................T..........A................................................................................................................A.....................

>Montaña3.2_79 .............................C...............A...........................................A..........................................................................................

>MC3.2Cons1 GACACAATACATACCCCTCACGCGGATTATCTCTCTCCACTCCCCGGCCGGAACACAAGCACCCTTAAGCCCAATAGTCCCTAGTACCAGAAACTATCTGTCCTCGTACTGAAACCTACCTAGCTCTTTCTCTGCACACCCCAAACCTTCCTAGATACGGATATACTCAACCACCCAAAA

>MC3.2Cons2 GACACAATACATACCCCTCACGCGGATTATCTCTTTCCACTCCCCAGCCGGAACACAAGCACCCTTAAGCCCAATAGTCCCTAGTACCAAAAACTATCTGTCCTCGTACTGAAACCTACCTAGCTCTTTCTCTGCACACCCCAAACCTTCCTAGATACGGATATACTCAACCACCCAAAA

**Pool: Montaña4**

>FJ755614H132 CAAACACATTCAATGCATGGACACAATACATACCTCTCACGCGGATTATCTCCCTCCACTCCCCGGCCGGAACACAAGCACCCTTAAGCCCAATAGTCCCTAGTACCAGAAACTATCTGTCCTCGTACTGAAACCTACCTAACTCTTTCTCTGCACACCTCAAATCCTCCTAGATACGGATATACTCAACCACCCAAAATCAATCGTAGCAGGACAAAT

>Montaña4_63 ..............................................................................................C.....................................................................................

>Montaña4_58 ...............................C.T..TT........................................................C.....................................................................................

>Montaña4_64 ..............................................................................................C.............................................C....C.T................................

>Montaña4_59 ..............................................................................................C.............................................C....C.T................................

>Montaña4_24 CAAACACATTCAATGCATG..............................................................................................C.............................................C....C.T................................TCAATCGTAGCAGGACAAAT

>Montaña4_22 ......................................................................................T.......C.....................................................................................

>Montaña4_58 CAAACACATTTAATGCATG...............................C.T...T......................................GTCCCTAGTACCAGAAAC

>Montaña4_61 ............................................................................

>Montaña4_37 ...............................C.T...T......................................

>Montaña4_49 ...............................C.T...T......................................

>Montaña4_59 ...............................C.T...T...............T......................

>Montaña4_43 ..............T.T..............C.T...T......................................

>Montaña4_42 ...............................C.T...T......................................

>Montaña4_41 ...............................C.T...T......................................

>Montaña4_44 ...............................C.T...T......................................

>Montaña4_62 ............................................................................

>Montaña4_53 .............T..T...........................................................

>Montaña4_48 ...................................................G.....A..................

>Montaña4_39 ............................................................................

>Montaña4_45 ...................................................G.G..............A.......

>Montaña4_54 A..............................C.T...T.T.....A..............................

>Montaña4_46 ............................................................................

>Montaña4_38 .................................T...T.......................T..............

>Montaña4_22 CAAACACATTTAATGCATG...............................................................T............GTCCCTAGTACCAGAAAC

>Montaña4_20 ............................................................................

>Montaña4_19 ...............................C.T...T...........A..........................

>Montaña4_25 ...............................C.T...T...........A..........................

>Montaña4_11 ...............................C.T...T......................................

>Montaña4_26 .................................T...T......................................

>Montaña4_14 .....................................................AG.....................

>Montaña4_18 ...............................C.T...T......................................

>Montaña4_12 ...............................C.TT..T......................................

>Montaña4_6 ...............................C.T...T......................................

>Montaña4_17 .......................................................................T....

>Montaña4_13 ............................................................................

>Montaña4_9 ...............................C.T...T.....T................................

>Montaña4_15 ............................................................................

>Montaña4_10 ...............................C.T...T......................................

>Montaña4_7 ..............T................C.T...T......................................

>Montaña4_H03 CCCTTAAGCCCAATAGTCC............................................................C....C.T................................TCAATCGTAGCAGGACAAA

>Montaña4_G03 ..........................................................N........N................................

>Montaña4_E03 ....................................................................................................

>Montaña4_B03 ...................................................................N................................

>Montaña4_C03 ...................................................................N................................

>Montaña4_A03 .......................................T....................C....C.T................................

>Montaña4_D03 ..............................A.....................................................................

>Montaña4_F03 ..............C............................................N........................................

>Montaña4_22 CCCTTAAGCCCAATAGTCC..............C.............................................C....C.T................................TCAATCGTAGCAGGACAAA

>Montaña4_3 ..............C.....................................................................................

>Montaña4_5 ..............C.............................................C....C.T........A.......................

>Montaña4_6 ..............C.....................................................................................

>Montaña4_2 ..............C.............................................C....C.T................................

>Montaña4_4 ..............C.............................................C....C.T................................

>Montaña4_7 ..............C.....................................................................................

>MC4Cons1 GACACAATACATACCTCTCACGCGGATTATCCCTCTCTACTCCCCGGCCGGAACACAAGCACCCTTAAGCCCAATAGTCCCTAGTACCAGAAACCATCTGTCCTCGTACTGAAACCTACCTAACTCTTTCTCTGCACACCTCAAATCCTCCTAGATACGGATATACTCAACCACCCAAAA

>MC4Cons2 GACACAATACATACCTCTCACGCGGATTATCTCCCTCCACTCCCCGGCCGGAACACAAGCACCCTTAAGCCCAATAGTCCCTAGTACCAGAAACCATCTGTCCTCGTACTGAAACCTACCTAACTCTTTCTCTGCACACCCCAAACCTTCCTAGATACGGATATACTCAACCACCCAAAA

**Pool: Montaña5**

>FJ755609H127 CAAACACATTCAATGCATGGACACAATACATACCTCTCACGCGGATTATCTCTCTCCACTCCCCGGCCGGAACACAAGCACCCTTAAGCCCAATAGTCCCTAGTACCAGAAACTATCTGTCCTCGTACTGAAACCTACCTAACTCTTTCTCTGCACACCTCAAATCCTCCTAGATACGGATATACTCAACCACCCAAAATCAATCGTAGCAGGACAAA

>Montaña5_23 ..................T..........................................T...................................................................T..................................................

>Montaña5_21 .................................................................C...............................................................T..................................................

>Montaña5_22 ....T....T.......................................................................................................................T........T...............................T.........

>Montaña5_17 .................................................................................................................................T.....................C...C...NN...................

>Montaña5_19 .................................................................................................................................T............................NNN...................

>Montaña5_20 .................................................................................................................................T..................................................

>Montaña5_24 .................................................................C...............................................................T..........CT...C.T............NN..................

>Montaña5_30 CAAACACATTCAATGCATG..................T..........................................T...................................................................T..................................................TCAATCGTAGCAGGACAAA

>Montaña5_28 .....................................................................T...........................................................T..................................................

>Montaña5_26 .................................................................C...........................................................T...T..........CT...C.T................................

>Montaña5_29 .................................................................C...............................................................T..........CT...C.T................................

>Montaña5_31 .................................................................C...............................................................T..........CT...C.T................................

>Montaña5_25 ..................T..............................................C...........................................................T...T..........CT...C.T................................

>Montaña5_G5 CAAACACATTCAATGCATG.............NN................N.................................C..........GTCCCTAGTACCAGAAAC

>Montaña5_A6 ............................................................................

>Montaña5_E7 ................................................................N...........

>Montaña5_C9 N..N...N.................................N......N...............N...........

>Montaña5_D8 ...............................................................N.C..........

>Montaña5_D9 ................................................N...............N...........

>Montaña5_B9 ................................................N...........................

>Montaña5_B6 ................................................N................C..........

>Montaña5_G6 ..........................................T.................................

>Montaña5_H5 .............................................................T...C..........

>Montaña5_A7 ..................................N.............N................C..........

>Montaña5_F7 ..........................................N......................C..........

>Montaña5_F8 ..................................N......TN.................................

>Montaña5_H7 ............................................................................

>Montaña5_H8 ............................N...............................................

>Montaña5_G7 ............................................................................

>Montaña5_A8 ..................................N.............N....N...........C..........

>Montaña5_C6 ................................................N...........................

>Montaña5_D7 ................................................N...........................

>Montaña5_D6 .................................................................C..........

>Montaña5_C7 ................................................N...........................

>Montaña5_C8 ............................................................................

>Montaña5_E6 ..................................N.............N................C..........

>Montaña5_B7 ................................................N...........................

>Montaña5_82 CAAACACATTCAATGCATG............................................................................GTCCCTAGTACCAGAAAC

>Montaña5_83 ............................................................................

>Montaña5_79 ............................................................................

>Montaña5_86 .......................................T....................................

>Montaña5_84 ....T.......................................................................

>Montaña5_73 .................................................................C..........

>Montaña5_92 ...............................................................T.C..........

>Montaña5_66 ............................................................................

>Montaña5_75 ............................................................................

>Montaña5_71 .................................................................C..........

>Montaña5_91 ............................................................................

>Montaña5_81 .............................................................TT.............

>Montaña5_69 .................................................................C..........

>Montaña5_70 ............................................................................

>Montaña5_76 ............................................................................

>Montaña5_74 ............................................................................

>Montaña5_90 ............................................................................

>Montaña5_68 ..................................................A.....G...................

>Montaña5_78 .................................................................C..........

>Montaña5_70 CAAACACATTCAATGCATG............................................................................GTCCCTAGTACCAGAAAC

>Montaña5_78 ............................................................................

>Montaña5_68 .............T..............................................................

>Montaña5_66 .......................................................T....................

>Montaña5_76 ............................................................................

>Montaña5_71 ............................................................................

>Montaña5_74 ............................................................................

>Montaña5_79 ............................................................................

>Montaña5_65 .......................................................T....................

>Montaña5_77 ............................................................................

>Montaña5_67 ............................................................................

>Montaña5_75 ............................................................................

>Montaña5_72 ............................................................................

>Montaña5_69 ............................................................................

>Montaña5_73 ..............G.............................................................

>Montaña5_G11 CCCTTAAGCCCAATAGTCC.................................................T..........NN...N.N................................TCAATCGTAGCAGGACAAA

>Montaña5_E11 ................................................NT..........CN...CNTN...N...........................

>Montaña5_D10 .................................................T.................N................................

>Montaña5_E12 .................................................T..................................................

>Montaña5_A10 .................................................T..........CT...C.T................................

>Montaña5_B11 .................................................T..........CT...C.T................................

>Montaña5_B12 .................................................T..........NN...C.N................................

>Montaña5_C11 .................................................T..................................................

>Montaña5_G9 .................................................T........N...........N.............................

>Montaña5_F10 .................................................T..................................................

>Montaña5_C10 .................................................T..........CT...C.T................................

>Montaña5_A12 .................................................T..........CT...C.T.......NNN..TNNN................

>Montaña5_H12 .................................................T..........CT...C.T......NNNNNNNNN.................

>Montaña5_H9 .................................................T..........CT...C.T..............NNN...............

>Montaña5_D11 .................................................T..........CT...C.T................................

>Montaña5_B10 ...................................T.............T..................................................

>Montaña5_D12 .................................................T..................................................

>Montaña5_H10 .................................................T.................N........NNNNNNNN................

>Montaña5_F12 .................................................T..........CT...C.T................................

>Montaña5_A11 .................................................T..................................................

>Montaña5_74 CCCTTAAGCCCAATAGTCC.................................................T..................................................TCAATCGTAGCAGGACAAA

>Montaña5_75 .................................................T..........CT...C.T................................

>Montaña5_86 .................................................T.T................................................

>Montaña5_93 .................................................T..........CT...C.T..T.............................

>Montaña5_92 .................................................T........T.CT...C.T................................

>Montaña5_84 .................................................T..........CT...C.T................................

>Montaña5_82 .................................................T............................................T.....

>Montaña5_65 .................................................T..................................................

>Montaña5_69 ..............C..................................T..................................................

>Montaña5_67 .................................................T...A..............................................

>Montaña5_77 .................................................T..........CT..GC.T................................

>Montaña5_85 .................................................T..................................................

>Montaña5_66 .................................................T..................................................

>Montaña5_73 ......................................T..........T..................................................

>Montaña5_83 .................................................T..........CT...C.C................................

>Montaña5_68 .................................................T..................................................

>Montaña5_76 .................................................T..................................................

>Montaña5_G02 CCCTTAAGCCCAATAGTCC.................................................T..................................................TCAATCGTAGCAGGACAAA

>Montaña5_G01 .................................................T..................................................

>MC5Cons1 GACACAATACATACCTCTCACGCGGATTATCTCTCTCCACTCCCCGGCCGGAACACAAGCACCCTTAAGCCCAATAGTCCCTAGTACCAGAAACTATCTGTCCTCGTACTGAAACCTACCTAACTCTTTTTCTGCACACCTCAAATCCTCCTAGATACGGATATACTCAACCACCCAAAA

>MC5Cons2 GACACAATACATACCTCTCACGCGGATTATCTCTCTCCACTCCCCGGCCGGAACACAAGCACCCTCAAGCCCAATAGTCCCTAGTACCAGAAACTATCTGTCCTCGTACTGAAACCTACCTAACTCTTTTTCTGCACACCCTAAACCTTCCTAGATACGGATATACTCAACCACCCAAAA

**Pool: Montaña5.2**

>FJ755609H127 CAAACACATTCAATGCATGGACACAATACATACCTCTCACGCGGATTATCTCTCTCCACTCCCCGGCCGGAACACAAGCACCCTTAAGCCCAATAGTCCCTAGTACCAGAAACTATCTGTCCTCGTACTGAAACCTACCTAACTCTTTCTCTGCACACCTCAAATCCTCCTAGATACGGATATACTCAACCACCCAAAATCAATCGTAGCAGGACAAA

>Montaña5.2_52 ....................................................................................................................................................................................

>Montaña5.2_63 A...........................................................................................................................................CT...C.T................................

>Montaña5.2_65 A.......................................................................................................................................T...........................................

>Montaña5.2_58 A...................................................................................................................................................................................

>Montaña5.2_56 ....................................................................................................................................................................................

>Montaña5.2_68 ....................................TT..............................................................................................................................................

>Montaña5.2_50 A...........................................................................................................................................CT...C.T................................

>Montaña5.2_54 A...........................................................................................................................................CT...C.T................................

>Montaña5.2_60 ....................................................................................................................................................................................

>Montaña5.2_49 ....................................................................................................................................................................................

>Montaña5.2_64 ....................................TT..............................................................................................................................................

>Montaña5.2_55 ...............................C....TT..............................................................................................................................................

>Montaña5.2_61 A....................................T..............................................................................................................................................

>Montaña5.2_57 ............................................................................................................................................CT...C.T................................

>Montaña5.2_67 ....................................TT......................................................................................................CT...C.T................................

>Montaña5.2_62 A...........................................................................................................................................CT...C.T................................

>Montaña5.2_53 ....................................................................................................................................................................................

>Montaña5.2_51 ....................................TT..............................................................................................................................................

>Montaña5.2_H6 CAAACACATTCAATGCATG....................................TT...........A...............................................................................T..................................................TCAATCGTAGCAGGACAAA

>Montaña5.2_H4 ....................................TT...........................................................................................T..................................................

>Montaña5.2_D6 ....................................TT...........................................................................................T..........C....C.T................................

>Montaña5.2_D5 ....................................TT...TT.................................................................................................C....C.T................................

>Montaña5.2_C4 .............T......................TT......T.......................................................................................................................................

>Montaña5.2_A5 ....................................TT...........................................................................................T..................................................

>Montaña5.2_G5 ....................................TT........................................................................A..................T..................................................

>Montaña5.2_E6 ....................................TT...........................................................................................T...............C..................................

>Montaña5.2_C5 ....................................TT...........................................................................................T..................................................

>Montaña5.2_A4 ....................................TT............T........T.T................T..................................................T..................................................

>Montaña5.2_C6 ....................................TT......................................................................................................C....C.T................................

>Montaña5.2_H5 ....................................TT...........................................................................................T..................................................

>Montaña5.2_D4 .............................C......TT...........................................................................................T..................................................

>Montaña5.2_F4 ....................................TT......................................................................................................C....C.T................................

>Montaña5.2_F6 ....................T...............TT...........................................................................................T..................................................

>Montaña5.2_B4 ....................................TT......................................................................................................C....C.T................................

>Montaña5.2_B5 ....................................TT...........................................................................................T..................................................

>Montaña5.2_B6 ....................................TT...........................................................................................T..................................................

>Montaña5.2_E5 ....................................TT...........................................................................................T..................................................

>Montaña5.2_G6 ....................................TT...........................................................................................T..................................................

>Montaña5.2_G4 ....................................TT...........................................................................................T..................................................

>Montaña5.2_F5 ....................................TT...........................................................................................T..................................................

>Montaña5.2_A6 ....................................TT.....................................................................................................TC....C.T................................

>Montaña5.2_35 CAAACACATTCAATGCATG....................................TT...........................................................................................T..................................................TCAATCGTAGCAGGACAAA

>Montaña5.2_41 ....................................TT...........................................................................................T.................T................................

>Montaña5.2_34 ....................................TT...........................................................................................T..................................................

>Montaña5.2_44 ....................................TT...........................................................................................T..................................................

>Montaña5.2_43 ....................................TT...........................................................................................T..................................................

>Montaña5.2_36 ....................................TT...........................................................................................T..................................................

>Montaña5.2_45 ....................................TT......................................................................................................C....C.T................................

>Montaña5.2_40 ....T...............................TT...........................................................................................T..................................................

>Montaña5.2_47 ....................................TT...........................................................................................T..................................................

>Montaña5.2_38 ....................................TT..............................................................................................................................................

>Montaña5.2_37 ....................................TT...........................................................................................T..................................................

>Montaña5.2_46 ....................................TT...........................................................................................T..................................................

>Montaña5.2_48 ................T...................TT......................................................................................................C....C.T................................

>Montaña5.2_4 CAAACACATTCAATGCATG....................................................................................................................................................................................TCAATCGTAGCAGGACAAA

>Montaña5.2_5 ....................................................................................................................................................................................

>Montaña5.2_7 ....................................................................................................................................................................................

>Montaña5.2_8 ................T...............T...................................................................................................................................................

>Montaña5.2_9 ....................................................................................................................................................................................

>Montaña5.2_4 CAAACACATTCAATGCATG....................................TT......................................................................................................C....C.T................................TCAATCGTAGCAGGACAAA

>Montaña5.2_5 ....................................TT......................................................................................................C....C.T................................

>Montaña5.2_7 ....................................TT...........................................................................................T..................................................

>Montaña5.2_8 ................T...............T...TT...........................................................................................T..................................................

>Montaña5.2_9 .....................................T...........................................................................................T.......................A..........................

>MC5.2Cons1 GACACAATACATACCTCTCACGCGGATTATCTCTCTTTACTCCCCGGCCGGAACACAAGCACCCTTAAGCCCAATAGTCCCTAGTACCAGAAACTATCTGTCCTCGTACTGAAACCTACCTAACTCTTTTTCTGCACACCTCAAATCCTCCTAGATACGGATATACTCAACCACCCAAAA

>MC5.2Cons2 GACACAATACATACCTCTCACGCGGATTATCTCTCTTTACTCCCCGGCCGGAACACAAGCACCCTTAAGCCCAATAGTCCCTAGTACCAGAAACTATCTGTCCTCGTACTGAAACCTACCTAACTCTTTCTCTGCACACCCCAAACCTTCCTAGATACGGATATACTCAACCACCCAAAA

**Pool: Montaña6**

>FJ755561H79 CAAACACATTCAATGCATGGACACAATACATACCTCTCACGCGGATTATCTCTCTCCACTCCCCGGCCGGAACACAAGCACCCTTAAGCCCAATAGTCCCTAGTACCAGAAACTATCTGTCCTCGTACTGAAACCTACCTAACTCTTTCTCTGCACACCCTAAACCTTCCTAGATACGGATATACTCAACCACCCAAAATCAATCGTAGCAGGACAAA

>Montaña6_26 ..............................................................................................C............................................TTC...T.................G..........T.....

>Montaña6_29 ..T...........................................................................................C............................................TTC...T............................T.....

>Montaña6_28 ..............................................................................................C................................................................................T....

>Montaña6_31 ..................................................................................G...........C................................................................................T....

>Montaña6_30 ..............................................................................................C................................................................................T....

>Montaña6_27 ..............................................................................................C...............................................................................T.....

>Montaña6_25 ..............................................................................................C............................................TTC...T............................T.....

>Montaña6_32 ..............................................................................................C...............................................................................T.....

>Montaña6_G08 CAAACACATTCAATGCATG...............................................T..........A.................GTCCCTAGTACCAGAAAC

>Montaña6_A08 ............................................................................

>Montaña6_B08 ............................................................................

>Montaña6_G07 ............................................................................

>Montaña6_F07 ...............................C.....T.............................G........

>Montaña6_D07 ............................................................................

>Montaña6_F08 ............................................................................

>Montaña6_C07 ............................................................................

>Montaña6_E08 ............................................................................

>Montaña6_C08 ............................................................................

>Montaña6_E07 ............................................................................

>Montaña6_D08 ............................................................................

>Montaña6_B07 ............................................................................

>Montaña6_A07 ............................................................................

>Montaña6_H07 ..........................................................A.................

>Montaña6_3 CAAACACATTCAATGCATG.........................G..................................................GTCCCTAGTACCAGAAAC

>Montaña6_1 ............................................................................

>Montaña6_2 ............................................................................

>Montaña6_4 ................................................................C...........

>Montaña6_96 CCCTTAAGCCCAATAGTCC..............C..............................................C......................................TCAATCGTAGCAGGACAAA

>Montaña6_93 ..............C...............................................................................T.....

>Montaña6_89 ..............C................................................................................T....

>Montaña6_87 ..............C............................................TTC...T............................T.....

>Montaña6_81 ..............C................................................................................T....

>Montaña6_94 ..............C............................................TTC...T............................T.....

>Montaña6_82 ..............C.............................................T....T.C................................

>Montaña6_85 ..............C.................G..............................................................T....

>Montaña6_88 ..............C................................................................................T....

>Montaña6_92 ..............C.............................................T....T.C..........................T.....

>Montaña6_91 ..............C............................................TTC...TA...........................T.....

>Montaña6_90 ..............C.............................................T....T.............................T....

>Montaña6_9 CCCTTAAGCCCAATAGTCC..............C............................................TTC...T............................T.....TCAATCGTAGCAGGACAAA

>MC6Cons1 GACACAATACATACCTCTCACGCGGATTATCTCTCTCCACTCCCCGGCCGGAACACAAGCACCCTTAAGCCCAATAGTCCCTAGTACCAGAAACCATCTGTCCTCGTACTGAAACCTACCTAACTCTTTCTCTGCACACCCTAAACCTTCCTAGATACGGATATACTCAACCACCTAAAA

>MC6Cons2 GACACAATACATACCTCTCACGCGGATTATCTCTCTCCACTCCCCGGCCGGAACACAAGCACCCTTAAGCCCAATAGTCCCTAGTACCAGAAACCATCTGTCCTCGTACTGAAACCTACCTAACTCTTTCTCTGCACACTTCAAATCTTCCTAGATACGGATATACTCAACCACTCAAAA

**Pool: Montaña6.2**

>FJ755495H13 CAAACACATTCAATGCATGGACACAACACATACCTCCCACGCGGATTATCTCTCTCCACTCCCCGGCCGGAACACAAGCACCCTTAAGCCCAATAGTCCCTAGTACCAGAAACTATCTGTCCTCGTACTGAAACCTACCTAACTCTTTTTCTGCACACCTCAAATCCTCCTAGATACGGATATACTCAACCACCCAAAATCAATCGTAGCAGGACAAA

>Montaña6.2_53 .......T.......................................................T.............................................C......................................................................

>Montaña6.2_56 .......T.........T..............................................................G.............C....................T.......T.....C.........T.......T..........................T.....

>Montaña6.2_55 .......T.........T..........................................G....................................................................C..................................................

>Montaña6.2_54 .......T.......C.T..........................................G.......................................C............................C..................................................

>Montaña6.2_49 .......T.......C.T................T.........................G.......................................C............................C..................................................

>Montaña6.2_50 ......GT.........T................................................................GA..........C....................T.............C.........T.......T..........................T.....

>Montaña6.2_38 CAAACACATTCAATGCATG.......T.......C.T............................................................................C.....C............................C...........................A.A....................TCAATCGTAGCAGGACAAA

>Montaña6.2_39 .......T.........T............................................................................C....................T.............C.........T.......T..........................T.....

>Montaña6.2_36 .......T.........T............................................................................C....................T.............C.........T.......T..........................T.....

>Montaña6.2_34 .......T.........T............................................................................C....................T.............C.............................A....................

>Montaña6.2_37 .......T.........T............................................................................C....................T.............C.........T.......T..........................T.....

>Montaña6.2_40 .......T.......C.T..........................................G.......................................C............................C.............................A....................

>Montaña6.2_33 .......T.........T............................................................................C....................T.............C.........T.......T..........................T.....

>Montaña6.2_35 .......T.........T............................................................................C....................T.............C.........T.......T..........................T.....

>Montaña6.2_23 CAAACACATTCAATGCATG.......T.......C.T..........................................G...............GTCCCTAGTACCAGAAAC

>Montaña6.2_25 .......T........TT...........C..............................G...............

>Montaña6.2_27 .......T........TT..........................................G...............

>Montaña6.2_24 .......T.......C.T............................A...A.........G...............

>Montaña6.2_26 .......T........TT..........................................G...............

>Montaña6.2_30 .......T.......C.T..........................................G...............

>Montaña6.2_28 .......T.......C.T..........................................G...............

>Montaña6.2_22 .......T.......C.T............................A...A.........G...............

>Montaña6.2_29 .......T.......C.T..........................................G...............

>Montaña6.2_1 CCCTTAAGCCCAATAGTCC.....................T...........................C...........C......................................TCAATCGTAGCAGGACAAA

>Montaña6.2_2 ....................C............................C..C.......C....C.T................................

>MC6.2Cons1 GACACAATACATACCTCTCACGCGGATTATCTCTCTCCACTCCCCGGCCGGAACACAAGCACCCTTAAGCCCAATAGTCCCTAGTACCAGAAACCATCTGTCCTCGTACTGAAACTTACCTAACTCTTTCTCTGCACACTTCAAATCTTCCTAGATACGGATATACTCAACCACTCAAAA

>MC6.2Cons2 GACACAATACATACCCCTCACGCGGATTATCTCTCTCCACTCCCCGGCCGGAACACAAGCGCCCTTAAGCCCAATAGTCCCTAGTACCAGAAACTATCTGCCCTCGTACTGAAACCTACCTAACTCTTTCTCTGCACACCTCAAATCCTCCTAGATACGGATATACTCAACCACCCAAAA

**Pool: Montaña7**

>FJ755614H132 CAAACACATTCAATGCATGGACACAATACATACCTCTCACGCGGATTATCTCCCTCCACTCCCCGGCCGGAACACAAGCACCCTTAAGCCCAATAGTCCCTAGTACCAGAAACTATCTGTCCTCGTACTGAAACCTACCTAACTCTTTCTCTGCACACCTCAAATCCTCCTAGATACGGATATACTCAACCACCCAAAATCAATCGTAGCAGGACAAA

>Montaña7_37 ..............................................................................................C.....C............................................C..................................

>Montaña7_33 ...............................C.T..TT........................................................C.....C.......................................C....C.T................................

>Montaña7_39 ..............................................................................................C.....C............................................C..................................

>Montaña7_38 ................................T.............................................................C.....C............................................C..................................

>Montaña7_36 ..............................................................................................C.....C............................................C..................................

>Montaña7_34 ...............................C.T..TT........................................................C.....C.......................................C....C.T................................

>Montaña7_40 ..............................................................................................C.....C............................................C..................................

>Montaña7_B10 CAAACACATTCAATGCATG...............................C.T...T......................................GTCCCTAGTACCAGAAAC

>Montaña7_H10 ............................................................................

>Montaña7_F10 .....................A......................................................

>Montaña7_D09 .................................T.CTT......................................

>Montaña7_E09 ............................................................................

>Montaña7_E10 ...................G...........C.T...T......T..........T.............T......

>Montaña7_G09 ...............................C.T...T......................................

>Montaña7_C09 ............................................................................

>Montaña7_D10 ......................G........C.T...T......T..........T.............T......

>Montaña7_C10 ............................................................................

>Montaña7_A09 ............................................................................

>Montaña7_F09 ............................................................................

>Montaña7_B09 .................................T..TT......................................

>Montaña7_G10 ............................................................................

>Montaña7_A10 ...............................C.T...T......................................

>Montaña7_H09 ..........................................................A.................

>Montaña7_30 CAAACACATTCAATGCATG.................................T..........................................GTCCCTAGTACCAGAAAC

>Montaña7_31 A..T...C.........................T..........................................

>Montaña7_27 ................................AT...T.T..T.T..T.....T.T....................

>Montaña7_29 .................C.............C.T...T......................................

>Montaña7_68 CCCTTAAGCCCAATAGTCC..............C.....C.......................................C....C.T................................ TCAATCGTAGCAGGACAAA

>Montaña7_64 ..............C.....C.T.....................................C....C.T................................

>Montaña7_79 ..............C.....C.......................................C....C.T................................

>Montaña7_65 ..............C.....C.......................................C....C.T................................

>Montaña7_78 ..............C.....C............................................C..................................

>Montaña7_67 ..............C.....C............................................C..................................

>Montaña7_76 ....................CTT.....................................C....C.T................................

>Montaña7_62 ..............C.....C............................................C..................................

>Montaña7_75 ..............C.....C............................................C..................................

>Montaña7_71 ..............C.....C............................................C....T.............................

>Montaña7_63 ..............C.....C............................................C..................................

>Montaña7_69 ..............C.....C.......................................C....C.T.........................T......

>Montaña7_73 ..............C.....C.......................................C....C.T................................

>Montaña7_72 ..............C.....C............................................C..................................

>Montaña7_70 ..............C.....C.......................................C....C.T................................

>Montaña7_74 ..............C.....C.......................................C....C.T................................

>Montaña7_80 ..............C.....C............................................C..................................

>MC7Cons1 GACACAATACATACCTCTCACGCGGATTATCTCCCTCCACTCCCCGGCCGGAACACAAGCACCCTTAAGCCCAATAGTCCCTAGTACCAGAAACCATCTGCCCTCGTACTGAAACCTACCTAACTCTTTCTCTGCACACCTCAAACCCTCCTAGATACGGATATACTCAACCACCCAAAA

>MC7Cons2 GACACAATACATACCTCTCACGCGGATTATCCCTCTCTACTCCCCGGCCGGAACACAAGCACCCTTAAGCCCAATAGTCCCTAGTACCAGAAACCATCTGCCCTCGTACTGAAACCTACCTAACTCTTTCTCTGCACACCCCAAACCTTCCTAGATACGGATATACTCAACCACCCAAAA

**Pool: Montaña24**

>FJ755565H83 CAAACACATTCAATGCATGGACACAATACATACCTCTCACGCGGATTATCTCTCTCCACTCCCCGGCCGGAACACAGGCACCCTTAAGCCCAATAGTCCCTAGTACCAGAAACTATCTGTCCTCGTACTGAAACCTACCTAACTCTTTCTCTGCACACCCTAAACCTTCCTAGATACGGATATACTCAACCACCCAAAATCAATCGTAGCAGGACAAA

>Montaña24_48 ...................................................................................A................................................................................................

>Montaña24_47 .................................................................................GG.........................................................TC...T.C................................

>Montaña24_42 .................................................................................G.A................................................................................................

>Montaña24_45 ...................................................................................A........................................................TC...T.C................................

>Montaña24_44 ............................................................................................................................................TC...T.C................................

>Montaña24_43 ............................................................................................................................................TC...T.C................................

>Montaña24_46 ............................................................................................................................................TC...T.C................................

>Montaña24_47 CAAACACATTCAATGCATG...................................................................................A................................................................................................TCAATCGTAGCAGGACAAA

>Montaña24_43 ...................................................................................A................................................................................................

>Montaña24_46 .....................................................................................G..............................................................................................

>Montaña24_44 ........................................................................................................................................... TC...T.C................................

>Montaña24_42 ................................................................................................C...........................................TC...T.C................................

>Montaña24_41 ...................................................................................A................................................................................................

>Montaña24_48 ....................................................................................................................................................................................

>Montaña24_45 ...................................................................................A................................................................................................

>Montaña24_B11CAAACACATTCAATGCATG.........................................................A..................GTCCCTAGTACCAGAAAC

>Montaña24_E11 ............................................................................

>Montaña24_C11 ............................................................................

>Montaña24_F11 ..............................................A.............................

>Montaña24_G11 ............................................................................

>Montaña24_F12 ..............................T...T.T.......................................

>Montaña24_G12 .........................................................A..................

>Montaña24_E12 ............................................................................

>Montaña24_H11 ............................................................................

>Montaña24_C12 ............................................................................

>Montaña24_B12 ............................................................................

>Montaña24_A11 ............................................................................

>Montaña24_D11 .........................................................A..................

>Montaña24_D12 ............................................................................

>Montaña24_H12 .........................................................AA.................

>Montaña24_A12 .........................................................c..................

>montaña24_47 CCCTTAAGCCCAATAGTCC.C.A.................T..............................................................................TCAATCGTAGCAGGACAAA

>montaña24_39 ...A................................................................................................

>montaña24_52 ...A................................................................................................

>montaña24_43 ............................................................TC...T.C................................

>montaña24_50 ...A................................................................................................

>montaña24_53 ...A................................................................................................

>montaña24_46 ...A................................................................................................

>montaña24_55 ......T.....................................................TC...T.C................................

>montaña24_37 ...A..............................................................................G.................

>montaña24_57 ...A................................................................................................

>montaña24_45 ....................................................................................................

>montaña24_48 ............................................................TC...T.C................................

>montaña24_33 ............................................................TC...T.C................................

>montaña24_35 ...A................................................................................................

>montaña24_38 ............................................................TC...T.C................................

>montaña24_58 ...A................................................................................................

>montaña24_56 ...A..................................................................................C.............

>montaña24_34 ...A..................................................................................C.............

>montaña24_41 ............................................................TC...T.C................................

>Montaña24_16 ............................................................TC...T.C...C............................

>Montaña24_14 CCCTTAAGCCCAATAGTCC..................................T...T..........T..........TC...TTC..T........................T..G.TCAATCGTAGCAGGACAAA

>MC24Cons1 GACACAATACATACCTCTCACGCGGATTATCTCTCTCCACTCCCCGGCCGGAACACAGGCACCCTTAAGCCCAATAGTCCCTAATACCAGAAACTATCTGTCCTCGTACTGAAACCTACCTAACTCTTTCTCTGCACACCCTAAACCTTCCTAGATACGGATATACTCAACCACCCAAAA

>MC24Cons2 GACACAATACATACCTCTCACGCGGATTATCTCTCTCCACTCCCCGGCCGGAACACAGGCACCCTTAAGCCCAATAGTCCCTAGTACCAGAAACTATCTGTCCTCGTACTGAAACCTACCTAACTCTTTCTCTGCACACCTCAAATCCTCCTAGATACGGATATACTCAACCACCCAAAA
