# Supplementary material for: Population connectivity buffers genetic diversity loss in a seabird
Source: Front Zool. 2013 May 20;10:28. doi: 10.1186/1742-9994-10-28 (PMC3662614; doi:10.1186/1742-9994-10-28)
Supplement: Additional file 3: Table S2 — Calonectris specimens from the extant and the extinct populations included in this study (307 in total). The majority of the modern samples (240 out of 282); are part of a genetic study previously published by the authors [16]. The GenBank accession numbers, as well as the geographic origin for each individual sequence, are indicated. [file 1742-9994-10-28-S3.docx]

**Table S1.** Conventional radiocarbon ages (yr BP) and 2σ calibration intervals (cal AD) from 3 bones of Cory`s Shearwater (*Calonectris borealis*) from Montaña Clara Islet (Canary Islands). The lower value of each interval must be considered a maximum age of each bone.

| Lab number | Radiocarbon age yr BP | 2σ calibration interval cal AD |
| --- | --- | --- |
| LuS 6428 | 1305 ± 60 | 1004 - 1451 |
| LuS 6427 | 1190 ± 50 | 1078 - 1530 |
| LuS 6429 | 775 ± 50 | *1499 → |

*The 2σ calibration interval of this sample may extend out of the calibration range.
