# Supplementary material for: Population connectivity buffers genetic diversity loss in a seabird
Source: Front Zool. 2013 May 20;10:28. doi: 10.1186/1742-9994-10-28 (PMC3662614; doi:10.1186/1742-9994-10-28)
Supplement: Additional file 4 — Supplementary Methods. [file 1742-9994-10-28-S4.docx]

**Table S2.***Calonectris* specimens from the extant and the extinct populations included in this study (307 in total)*.* The majority of the modern samples (240 out of 282); are part of a genetic study previously published by the authors (Gómez-Díaz et al. 2009). The GenBank accession numbers, as well as the geographic origin for each individual sequence, are indicated.

| **SPECIES** | **ID CODE** | **Geographic Locality** | **GenBank Ac.No** | **Dataset** |
| --- | --- | --- | --- | --- |
| ***Calonectris diomedea*** | 6114296 | Chafarinas Is.- Morocco N coast | FJ755483 | Gómez-Díaz *et al.* 2009 |
| *C. diomedea* | 6114393 | Chafarinas Is.- Morocco N coast | FJ755484 | Gómez-Díaz *et al.* 2009 |
| *C. diomedea* | 6114410 | Chafarinas Is.- Morocco N coast | FJ755485 | Gómez-Díaz *et al.* 2009 |
| *C. diomedea* | 6114444 | Chafarinas Is.- Morocco N coast | FJ755486 | Gómez-Díaz *et al.* 2009 |
| *C. diomedea* | 6114445 | Chafarinas Is.- Morocco N coast | FJ755487 | Gómez-Díaz *et al.* 2009 |
| *C. diomedea* | 6114450 | Chafarinas Is.- Morocco N coast | FJ755488 | Gómez-Díaz *et al.* 2009 |
| *C. diomedea* | 6114651 | Chafarinas Is.- Morocco N coast | FJ755489 | Gómez-Díaz *et al.* 2009 |
| *C. diomedea* | 6114653 | Chafarinas Is.- Morocco N coast | FJ755490 | Gómez-Díaz *et al.* 2009 |
| *C. diomedea* | 6114655 | Chafarinas Is.- Morocco N coast | FJ755491 | Gómez-Díaz *et al.* 2009 |
| *C. diomedea* | 6120516 | Chafarinas Is.- Morocco N coast | FJ755492 | Gómez-Díaz *et al.* 2009 |
| *C. diomedea* | 6031150 | Pantaleu- Mallorca | FJ755493 | Gómez-Díaz *et al.* 2009 |
| *C. diomedea* | 6048668 | Pantaleu- Mallorca | FJ755494 | Gómez-Díaz *et al.* 2009 |
| *C. diomedea* | 6073389 | Pantaleu- Mallorca | FJ755495 | Gómez-Díaz *et al.* 2009 |
| *C. diomedea* | 6123028 | Pantaleu- Mallorca | FJ755496 | Gómez-Díaz *et al.* 2009 |
| *C. diomedea* | 6129219 | Pantaleu- Mallorca | FJ755497 | Gómez-Díaz *et al.* 2009 |
| *C. diomedea* | 6131482 | Pantaleu- Mallorca | FJ755498 | Gómez-Díaz *et al.* 2009 |
| *C. diomedea* | 6140103 | Pantaleu- Mallorca | FJ755499 | Gómez-Díaz *et al.* 2009 |
| *C. diomedea* | 3202 | Toro- Mallorca- Balearic Is. | FJ755483 | Gómez-Díaz *et al.* 2009 |
| *C. diomedea* | 3203 | Toro- Mallorca- Balearic Is. | FJ755500 | Gómez-Díaz *et al.* 2009 |
| *C. diomedea* | 3204 | Toro- Mallorca- Balearic Is. | FJ755494 | Gómez-Díaz *et al.* 2009 |
| *C. diomedea* | 3205 | Toro- Mallorca- Balearic Is. | FJ755501 | Gómez-Díaz *et al.* 2009 |
| *C. diomedea* | T72388 | Linosa- Italy | FJ755483 | Gómez-Díaz *et al.* 2009 |
| *C. diomedea* | T72742 | Linosa- Italy | FJ755502 | Gómez-Díaz *et al.* 2009 |
| *C. diomedea* | T73703 | Linosa- Italy | FJ755503 | Gómez-Díaz *et al.* 2009 |
| *C. diomedea* | T73706 | Linosa- Italy | FJ755503 | Gómez-Díaz *et al.* 2009 |
| *C. diomedea* | T73708 | Linosa- Italy | FJ755504 | Gómez-Díaz *et al.* 2009 |
| *C. diomedea* | T73711 | Linosa- Italy | FJ755505 | Gómez-Díaz *et al.* 2009 |
| *C. diomedea* | T73714 | Linosa- Italy | FJ755506 | Gómez-Díaz *et al.* 2009 |
| *C. diomedea* | T73717 | Linosa- Italy | FJ755503 | Gómez-Díaz *et al.* 2009 |
| *C. diomedea* | T73719 | Linosa- Italy | FJ755507 | Gómez-Díaz *et al.* 2009 |
| *C. diomedea* | T90126 | Sardinia- Italy | FJ755508 | Gómez-Díaz *et al.* 2009 |
| *C. diomedea* | T90127 | Sardinia- Italy | FJ755509 | Gómez-Díaz *et al.* 2009 |
| *C. diomedea* | T90128 | Sardinia- Italy | FJ755503 | Gómez-Díaz *et al.* 2009 |
| *C. diomedea* | T90129 | Sardinia- Italy | FJ755510 | Gómez-Díaz *et al.* 2009 |
| *C. diomedea* | TA2140 | Tuscany- Italy | FJ755511 | Gómez-Díaz *et al.* 2009 |
| *C. diomedea* | TA2141 | Tuscany- Italy | FJ755498 | Gómez-Díaz *et al.* 2009 |
| *C. diomedea* | TA2142 | Tuscany- Italy | FJ755494 | Gómez-Díaz *et al.* 2009 |
| *C. diomedea* | TA2143 | Tuscany- Italy | FJ755502 | Gómez-Díaz *et al.* 2009 |
| *C. diomedea* | TA2144 | Tuscany- Italy | FJ755512 | Gómez-Díaz *et al.* 2009 |
| *C. diomedea* | TA2145 | Tuscany- Italy | FJ755494 | Gómez-Díaz *et al.* 2009 |
| *C. diomedea* | 6010846 | Palomas Is.- Spain E coast | FJ755513 | Gómez-Díaz *et al.* 2009 |
| *C. diomedea* | 6010995 | Palomas Is.- Spain E coast | FJ755514 | Gómez-Díaz *et al.* 2009 |
| *C. diomedea* | 6058129 | Palomas Is.- Spain E coast | FJ755515 | Gómez-Díaz *et al.* 2009 |
| *C. diomedea* | 6140439 | Palomas Is.- Spain E coast | FJ755514 | Gómez-Díaz *et al.* 2009 |
| *C. diomedea* | 6140441 | Palomas Is.- Spain E coast | FJ755516 | Gómez-Díaz *et al.* 2009 |
| *C. diomedea* | 6149120 | Palomas Is.- Spain E coast | FJ755518 | Gómez-Díaz *et al.* 2009 |
| *C. diomedea* | 6149121 | Palomas Is.- Spain E coast | FJ755514 | Gómez-Díaz *et al.* 2009 |
| *C. diomedea* | 6149125 | Palomas Is.- Spain E coast | FJ755501 | Gómez-Díaz *et al.* 2009 |
| *C. diomedea* | 6149127 | Palomas Is.- Spain E coast | FJ755514 | Gómez-Díaz *et al.* 2009 |
| *C. diomedea* | 6149128 | Palomas Is.- Spain E coast | FJ755501 | Gómez-Díaz *et al.* 2009 |
| *C. diomedea* | 6137530 | S.Salomó- Menorca- Balearic Is. | FJ755498 | Gómez-Díaz *et al.* 2009 |
| *C. diomedea* | 6137573 | S.Salomó- Menorca- Balearic Is. | FJ755494 | Gómez-Díaz *et al.* 2009 |
| *C. diomedea* | 6137574 | S.Salomó- Menorca- Balearic Is. | FJ755518 | Gómez-Díaz *et al.* 2009 |

Table S2 (continued)

| **SPECIES** | **ID CODE** | **Geographic Locality** | **GenBank Ac.No** | **Dataset** |
| --- | --- | --- | --- | --- |
| *C. diomedea* | 6140089 | Mola- Menorca- Balearic Is. | FJ755519 | Gómez-Díaz *et al.* 2009 |
| *C. diomedea* | 6140097 | Mola- Menorca- Balearic Is. | FJ755520 | Gómez-Díaz *et al.* 2009 |
| *C. diomedea* | 6140098 | Mola- Menorca- Balearic Is. | FJ755521 | Gómez-Díaz *et al.* 2009 |
| *C. diomedea* | 6140099 | Mola- Menorca- Balearic Is. | FJ755522 | Gómez-Díaz *et al.* 2009 |
| *C. diomedea* | 6037913 | Aire- Menorca- Balearic Is. | FJ755523 | Gómez-Díaz *et al.* 2009 |
| *C. diomedea* | 6137554 | Aire- Menorca- Balearic Is. | FJ755524 | Gómez-Díaz *et al.* 2009 |
| *C. diomedea* | 6137568 | Aire- Menorca- Balearic Is. | FJ755525 | Gómez-Díaz *et al.* 2009 |
| *C. diomedea* | 3319 | Na Pobre- Cabrera- Balearic Is. | FJ755506 | Gómez-Díaz *et al.* 2009 |
| *C. diomedea* | 3322 | Na Pobre- Cabrera- Balearic Is. | FJ755526 | Gómez-Díaz *et al.* 2009 |
| *C. diomedea* | 6076356 | Na Pobre- Cabrera- Balearic Is. | FJ755527 | Gómez-Díaz *et al.* 2009 |
| *C. diomedea* | 6076643 | Na Pobre- Cabrera- Balearic Is. | FJ755506 | Gómez-Díaz *et al.* 2009 |
| *C. diomedea* | 6127152 | Na Pobre- Cabrera- Balearic Is. | FJ755528 | Gómez-Díaz *et al.* 2009 |
| *C. diomedea* | 6127154 | Na Pobre- Cabrera- Balearic Is. | FJ755529 | Gómez-Díaz *et al.* 2009 |
| *C. diomedea* | 6143628 | Espartà- Eivissa- Balearic Is. | FJ755509 | Gómez-Díaz *et al.* 2009 |
| *C. diomedea* | 6143630 | Espartà- Eivissa- Balearic Is. | FJ755496 | Gómez-Díaz *et al.* 2009 |
| *C. diomedea* | 6143632 | Espartà- Eivissa- Balearic Is. | FJ755514 | Gómez-Díaz *et al.* 2009 |
| *C. diomedea* | 6143638 | Espartà- Eivissa- Balearic Is. | FJ755530 | Gómez-Díaz *et al.* 2009 |
| *C. diomedea* | 6143615 | Conillera- Eivissa- Balearic Is. | FJ755483 | Gómez-Díaz *et al.* 2009 |
| *C. diomedea* | 6143616 | Conillera- Eivissa- Balearic Is. | FJ755520 | Gómez-Díaz *et al.* 2009 |
| *C. diomedea* | 6143618 | Conillera- Eivissa- Balearic Is. | FJ755531 | Gómez-Díaz *et al.* 2009 |
| *C. diomedea* | 6143619 | Conillera- Eivissa- Balearic Is. | FJ755483 | Gómez-Díaz *et al.* 2009 |
| *C. diomedea* | 6086010 | Malvins- Eivissa- Balearic Is. | FJ755494 | Gómez-Díaz *et al.* 2009 |
| *C. diomedea* | 6086040 | Malvins- Eivissa- Balearic Is. | FJ755532 | Gómez-Díaz *et al.* 2009 |
| *C. diomedea* | 6086041 | Malvins- Eivissa- Balearic Is. | FJ755503 | Gómez-Díaz *et al.* 2009 |
| *C. diomedea* | 6143642 | Malvins- Eivissa- Balearic Is. | FJ755533 | Gómez-Díaz *et al.* 2009 |
| *C. diomedea* | 36573 | Tremiti – Italy | FJ755534 | Gómez-Díaz *et al.* 2009 |
| *C. diomedea* | 36574 | Tremiti – Italy | FJ755498 | Gómez-Díaz *et al.* 2009 |
| *C. diomedea* | 36586 | Tremiti – Italy | FJ755498 | Gómez-Díaz *et al.* 2009 |
| *C. diomedea* | 78623 | Tremiti – Italy | FJ755498 | Gómez-Díaz *et al.* 2009 |
| *C. diomedea* | 97738 | Tremiti – Italy | FJ755494 | Gómez-Díaz *et al.* 2009 |
| *C. diomedea* | 97756 | Tremiti – Italy | FJ755506 | Gómez-Díaz *et al.* 2009 |
| *C. diomedea* | 36579 | Tremiti- Italy | FJ755535 | Gómez-Díaz *et al.* 2009 |
| *C. diomedea* | 36591 | Tremiti- Italy | FJ755536 | Gómez-Díaz *et al.* 2009 |
| *C. diomedea* | 36592 | Tremiti- Italy | FJ755537 | Gómez-Díaz *et al.* 2009 |
| *C. diomedea* | EA537432 | Porquerolles Is. – France E coast | FJ755538 | Gómez-Díaz *et al.* 2009 |
| *C. diomedea* | EA537418 | Porquerolles Is. – France E coast | FJ755539 | Gómez-Díaz *et al.* 2009 |
| *C. diomedea* | EA537424 | Porquerolles Is. – France E coast | FJ755540 | Gómez-Díaz *et al.* 2009 |
| *C. diomedea* | EA537426 | Porquerolles Is. – France E coast | FJ755506 | Gómez-Díaz *et al.* 2009 |
| *C. diomedea* | EA537435 | Porquerolles Is. – France E coast | FJ755501 | Gómez-Díaz *et al.* 2009 |
| *C. diomedea* | EA537440 | Porquerolles Is. – France E coast | FJ755512 | Gómez-Díaz *et al.* 2009 |
| *C. diomedea* | EA537444 | Porquerolles Is. – France E coast | FJ755541 | Gómez-Díaz *et al.* 2009 |
| *C. diomedea* | EA537423 | Porquerolles Is. – France E coast | FJ755542 | Gómez-Díaz *et al.* 2009 |
| *C. diomedea* | EA537425 | Porquerolles Is.- France E coast | FJ755500 | Gómez-Díaz *et al.* 2009 |
| *C. diomedea* | EA572802 | Porquerolles Is.- France E coast | FJ755543 | Gómez-Díaz *et al.* 2009 |
| *C. diomedea* | 5301 | Creta- Greece | FJ755498 | Gómez-Díaz *et al.* 2009 |
| *C. diomedea* | 5303 | Creta- Greece | FJ755544 | Gómez-Díaz *et al.* 2009 |
| *C. diomedea* | 5305 | Creta- Greece | FJ755545 | Gómez-Díaz *et al.* 2009 |
| *C. diomedea* | 5306 | Creta- Greece | FJ755537 | Gómez-Díaz *et al.* 2009 |
| *C. diomedea* | 5311 | Creta- Greece | FJ755546 | Gómez-Díaz *et al.* 2009 |
| *C. diomedea* | 5316 | Creta- Greece | FJ755502 | Gómez-Díaz *et al.* 2009 |
| *C. diomedea* | 5319 | Creta- Greece | FJ755547 | Gómez-Díaz *et al.* 2009 |
| *C. diomedea* | 5329 | Creta- Greece | FJ755537 | Gómez-Díaz *et al.* 2009 |
| *C. diomedea* | MI353 | Creta- Greece | FJ755537 | Gómez-Díaz *et al.* 2009 |
| *C. diomedea* | 6059448 | Columbretes Is. – Spain E coast | FJ755503 | Gómez-Díaz *et al.* 2009 |
| *C. diomedea* | 6059449 | Columbretes Is. – Spain E coast | FJ755497 | Gómez-Díaz *et al.* 2009 |
| *C. diomedea* | 6059474 | Columbretes Is. – Spain E coast | FJ755548 | Gómez-Díaz *et al.* 2009 |
| *C. diomedea* | 6131707 | Columbretes Is. – Spain E coast | FJ755499 | Gómez-Díaz *et al.* 2009 |
| *C. diomedea* | 6131729 | Columbretes Is. – Spain E coast | FJ755518 | Gómez-Díaz *et al.* 2009 |
| *C. diomedea* | 6131732 | Columbretes Is. – Spain E coast | FJ755497 | Gómez-Díaz *et al.* 2009 |
| *C. diomedea* | 6131735 | Columbretes Is. – Spain E coast | FJ755489 | Gómez-Díaz *et al.* 2009 |

Table S2 (continued)

| **SPECIES** | **ID CODE** | **Geographic Locality** | **GenBank Ac.No** | **Dataset** |
| --- | --- | --- | --- | --- |
| *C. diomedea* | 6131736 | Columbretes Is. – Spain E coast | FJ755503 | Gómez-Díaz *et al.* 2009 |
| *C. diomedea* | 6131739 | Columbretes Is. – Spain E coast | FJ755549 | Gómez-Díaz *et al.* 2009 |
| *C. diomedea* | 6131741 | Columbretes Is.- Spain E coast | FJ755494 | Gómez-Díaz *et al.* 2009 |
| ***Calonectris borealis*** | L010192 | Vila-Santa Maria- Azores Is. | FJ755550 | Gómez-Díaz *et al.* 2009 |
| *C. borealis* | L010229 | Vila-Santa Maria- Azores Is. | FJ755551 | Gómez-Díaz *et al.* 2009 |
| *C. borealis* | L035254 | Vila-Santa Maria- Azores Is. | FJ755552 | Gómez-Díaz *et al.* 2009 |
| *C. borealis* | L038405 | Vila-Santa Maria- Azores Is. | FJ755553 | Gómez-Díaz *et al.* 2009 |
| *C. borealis* | L038434 | Vila-Santa Maria- Azores Is. | FJ755554 | Gómez-Díaz *et al.* 2009 |
| *C. borealis* | L038453 | Vila-Santa Maria- Azores Is. | FJ755555 | Gómez-Díaz *et al.* 2009 |
| *C. borealis* | L045670 | Vila-Santa Maria- Azores Is. | FJ755556 | Gómez-Díaz *et al.* 2009 |
| *C. borealis* | L045995 | Vila-Santa Maria- Azores Is. | FJ755557 | Gómez-Díaz *et al.* 2009 |
| *C. borealis* | L066676 | Vila-Santa Maria- Azores Is. | FJ755558 | Gómez-Díaz *et al.* 2009 |
| *C. borealis* | 6134562 | Veneguera- Gran Canaria- Canary Is. | FJ755552 | Gómez-Díaz *et al.* 2009 |
| *C. borealis* | 6140493 | Veneguera- Gran Canaria- Canary Is. | FJ755559 | Gómez-Díaz *et al.* 2009 |
| *C. borealis* | 6134502 | Gáldar-Gran Canaria- Canary Is. | FJ755560 | Gómez-Díaz *et al.* 2009 |
| *C. borealis* | 216134508 | Gáldar-Gran Canaria- Canary Is. | FJ755561 | Gómez-Díaz *et al.* 2009 |
| *C. borealis* | 6134503 | Gáldar- Gran Canaria- Canary Is. | FJ755560 | Gómez-Díaz *et al.* 2009 |
| *C. borealis* | 1101 | Vilafranca-Sao Miguel- Azores Is. | FJ755562 | Gómez-Díaz *et al.* 2009 |
| *C. borealis* | 1102 | Vilafranca-Sao Miguel- Azores Is. | FJ755556 | Gómez-Díaz *et al.* 2009 |
| *C. borealis* | 1103 | Vilafranca-Sao Miguel- Azores Is. | FJ755563 | Gómez-Díaz *et al.* 2009 |
| *C. borealis* | 1104 | Vilafranca-Sao Miguel- Azores Is. | FJ755564 | Gómez-Díaz *et al.* 2009 |
| *C. borealis* | 1107 | Vilafranca-Sao Miguel- Azores Is. | FJ755565 | Gómez-Díaz *et al.* 2009 |
| *C. borealis* | 1108 | Vilafranca-Sao Miguel- Azores Is. | FJ755563 | Gómez-Díaz *et al.* 2009 |
| *C. borealis* | L066680 | Vilafranca-Sao Miguel- Azores Is. | FJ755566 | Gómez-Díaz *et al.* 2009 |
| *C. borealis* | L066681 | Vilafranca-Sao Miguel- Azores Is. | FJ755567 | Gómez-Díaz *et al.* 2009 |
| *C. borealis* | L066682 | Vilafranca-Sao Miguel- Azores Is. | FJ755568 | Gómez-Díaz *et al.* 2009 |
| *C. borealis* | L066683 | Vilafranca-Sao Miguel- Azores Is. | FJ755569 | Gómez-Díaz *et al.* 2009 |
| *C. borealis* | 6134591 | Montaña Clara- Lanzarote- Canary Is. | FJ755552 | Gómez-Díaz *et al.* 2009 |
| *C. borealis* | 6134592 | Montaña Clara- Lanzarote- Canary Is. | FJ755570 | Gómez-Díaz *et al.* 2009 |
| *C. borealis* | 6134595 | Montaña Clara- Lanzarote- Canary Is. | FJ755557 | Gómez-Díaz *et al.* 2009 |
| *C. borealis* | 6134598 | Montaña Clara- Lanzarote- Canary Is. | FJ755571 | Gómez-Díaz *et al.* 2009 |
| *C. borealis* | 6134601 | Montaña Clara- Lanzarote- Canary Is. | FJ755557 | Gómez-Díaz *et al.* 2009 |
| *C. borealis* | 6134602 | Montaña Clara- Lanzarote- Canary Is. | FJ755552 | Gómez-Díaz *et al.* 2009 |
| *C. borealis* | 6134606 | Montaña Clara- Lanzarote- Canary Is. | FJ755572 | Gómez-Díaz *et al.* 2009 |
| *C. borealis* | 6134607 | Montaña Clara- Lanzarote- Canary Is. | FJ755573 | Gómez-Díaz *et al.* 2009 |
| *C. borealis* | 6134610 | Montaña Clara- Lanzarote- Canary Is. | FJ755574 | Gómez-Díaz *et al.* 2009 |
| *C. borealis* | 6134570 | Adeje –Tenerife- Canary Is. | FJ755545 | Gómez-Díaz *et al.* 2009 |
| *C. borealis* | 6134571 | Adeje –Tenerife- Canary Is. | FJ755557 | Gómez-Díaz *et al.* 2009 |
| *C. borealis* | 6134572 | Adeje –Tenerife- Canary Is. | FJ755552 | Gómez-Díaz *et al.* 2009 |
| *C. borealis* | 6134573 | Adeje -Tenerife- Canary Is. | FJ755575 | Gómez-Díaz *et al.* 2009 |
| *C. borealis* | 6134578 | Adeje -Tenerife- Canary Is. | FJ755552 | Gómez-Díaz *et al.* 2009 |
| *C. borealis* | 6134574 | Palm Mar- Tenerife- Canary Is. | FJ755575 | Gómez-Díaz *et al.* 2009 |
| *C. borealis* | 6134574 | Palm Mar- Tenerife- Canary Is. | FJ755552 | Gómez-Díaz *et al.* 2009 |
| *C. borealis* | 6134575 | Palm Mar- Tenerife- Canary Is. | FJ755552 | Gómez-Díaz *et al.* 2009 |
| *C. borealis* | 6134576 | Palm Mar- Tenerife- Canary Is. | FJ755576 | Gómez-Díaz *et al.* 2009 |
| *C. borealis* | 6134577 | Palm Mar- Tenerife- Canary Is. | FJ755553 | Gómez-Díaz *et al.* 2009 |
| *C. borealis* | L061802 | Praia- Graciosa- Azores Is. | FJ755566 | Gómez-Díaz *et al.* 2009 |
| *C. borealis* | L063061 | Praia- Graciosa- Azores Is. | FJ755577 | Gómez-Díaz *et al.* 2009 |
| *C. borealis* | L063092 | Praia- Graciosa- Azores Is. | FJ755565 | Gómez-Díaz *et al.* 2009 |
| *C. borealis* | L063100 | Praia- Graciosa- Azores Is. | FJ755578 | Gómez-Díaz *et al.* 2009 |
| *C. borealis* | L066692 | Praia- Graciosa- Azores Is. | FJ755579 | Gómez-Díaz *et al.* 2009 |
| *C. borealis* | L066693 | Praia- Graciosa- Azores Is. | FJ755557 | Gómez-Díaz *et al.* 2009 |
| *C. borealis* | L066696 | Praia- Graciosa- Azores Is. | FJ755561 | Gómez-Díaz *et al.* 2009 |
| *C. borealis* | L066700 | Praia- Graciosa- Azores Is. | FJ755561 | Gómez-Díaz *et al.* 2009 |
| *C. borealis* | L066701 | Praia- Graciosa- Azores Is. | FJ755580 | Gómez-Díaz *et al.* 2009 |
| *C. borealis* | L066702 | Praia- Graciosa- Azores Is. | FJ755553 | Gómez-Díaz *et al.* 2009 |
| *C. borealis* | L066703 | Moiño- Flores- Azores Is. | FJ755578 | Gómez-Díaz *et al.* 2009 |
| *C. borealis* | L066704 | Moiño- Flores- Azores Is. | FJ755581 | Gómez-Díaz *et al.* 2009 |
| *C. borealis* | L066706 | Lopo Vaz- Flores- Azores Is. | FJ755582 | Gómez-Díaz *et al.* 2009 |

Table S2 (continued)

| **SPECIES** | **ID CODE** | **Geographic Locality** | **GenBank Ac.No** | **Dataset** |
| --- | --- | --- | --- | --- |
| *C. borealis* | L066707 | Lopo Vaz- Flores- Azores Is. | FJ755552 | Gómez-Díaz *et al.* 2009 |
| *C. borealis* | L066708 | Lopo Vaz- Flores- Azores Is. | FJ755583 | Gómez-Díaz *et al.* 2009 |
| *C. borealis* | L066710 | Lopo Vaz- Flores- Azores Is. | FJ755584 | Gómez-Díaz *et al.* 2009 |
| *C. borealis* | L066712 | Lopo Vaz- Flores- Azores Is. | FJ755585 | Gómez-Díaz *et al.* 2009 |
| *C. borealis* | L066713 | Lopo Vaz- Flores- Azores Is. | FJ755582 | Gómez-Díaz *et al.* 2009 |
| *C. borealis* | L066716 | Lopo Vaz- Flores- Azores Is. | FJ755586 | Gómez-Díaz *et al.* 2009 |
| *C. borealis* | L066719 | Pesqueiros- Corvo- Azores Is. | FJ755556 | Gómez-Díaz *et al.* 2009 |
| *C. borealis* | L066720 | Pesqueiros- Corvo- Azores Is. | FJ755556 | Gómez-Díaz *et al.* 2009 |
| *C. borealis* | L066722 | Pesqueiros- Corvo- Azores Is. | FJ755557 | Gómez-Díaz *et al.* 2009 |
| *C. borealis* | L066723 | Pesqueiros- Corvo- Azores Is. | FJ755552 | Gómez-Díaz *et al.* 2009 |
| *C. borealis* | L066724 | Pesqueiros- Corvo- Azores Is. | FJ755587 | Gómez-Díaz *et al.* 2009 |
| *C. borealis* | L066732 | Pesqueiros- Corvo- Azores Is. | FJ755552 | Gómez-Díaz *et al.* 2009 |
| *C. borealis* | L066733 | Pesqueiros- Corvo- Azores Is. | FJ755554 | Gómez-Díaz *et al.* 2009 |
| *C. borealis* | L44205 | Pesqueiros- Corvo- Azores Is. | FJ755552 | Gómez-Díaz *et al.* 2009 |
| *C. borealis* | L44228 | Pesqueiros- Corvo- Azores Is. | FJ755570 | Gómez-Díaz *et al.* 2009 |
| *C. borealis* | L44260 | Pesqueiros- Corvo- Azores Is. | FJ755587 | Gómez-Díaz *et al.* 2009 |
| *C. borealis* | L066736 | Castelo Branco- Faial- Azores Is. | FJ755588 | Gómez-Díaz *et al.* 2009 |
| *C. borealis* | L066737 | Castelo Branco- Faial- Azores Is. | FJ755588 | Gómez-Díaz *et al.* 2009 |
| *C. borealis* | L066741 | Castelo Branco- Faial- Azores Is. | FJ755557 | Gómez-Díaz *et al.* 2009 |
| *C. borealis* | L066743 | Castelo Branco- Faial- Azores Is. | FJ755552 | Gómez-Díaz *et al.* 2009 |
| *C. borealis* | L066744 | Castelo Branco- Faial- Azores Is. | FJ755552 | Gómez-Díaz *et al.* 2009 |
| *C. borealis* | L066745 | Castelo Branco- Faial- Azores Is. | FJ755589 | Gómez-Díaz *et al.* 2009 |
| *C. borealis* | L066746 | Castelo Branco- Faial- Azores Is. | FJ755557 | Gómez-Díaz *et al.* 2009 |
| *C. borealis* | L011343 | Selvagems | FJ755575 | Gómez-Díaz *et al.* 2009 |
| *C. borealis* | L040031 | Selvagems | FJ755590 | Gómez-Díaz *et al.* 2009 |
| *C. borealis* | L046865 | Selvagems | FJ755591 | Gómez-Díaz *et al.* 2009 |
| *C. borealis* | L070026 | Selvagems | FJ755592 | Gómez-Díaz *et al.* 2009 |
| *C. borealis* | L070320 | Selvagems | FJ755593 | Gómez-Díaz *et al.* 2009 |
| *C. borealis* | L070601 | Selvagems | FJ755594 | Gómez-Díaz *et al.* 2009 |
| *C. borealis* | L43977 | Selvagems | FJ755595 | Gómez-Díaz *et al.* 2009 |
| *C. borealis* | 3501 | Terreros Is.- Spain E coast | FJ755557 | Gómez-Díaz *et al.* 2009 |
| *C. borealis* | 3503 | Terreros Is.- Spain E coast | FJ755563 | Gómez-Díaz *et al.* 2009 |
| *C. borealis* | 6107901 | Terreros Is.- Spain E coast | FJ755596 | Gómez-Díaz *et al.* 2009 |
| *C. borealis* | 6107903 | Terreros Is.- Spain E coast | FJ755597 | Gómez-Díaz *et al.* 2009 |
| *C. borealis* | 6107995 | Terreros Is.- Spain E coast | FJ755598 | Gómez-Díaz *et al.* 2009 |
| *C. borealis* | 6107997 | Terreros Is.- Spain E coast | FJ755599 | Gómez-Díaz *et al.* 2009 |
| *C. borealis* | 6108848 | Terreros Is.- Spain E coast | FJ755600 | Gómez-Díaz *et al.* 2009 |
| *C. borealis* | 6108849 | Terreros Is.- Spain E coast | FJ755552 | Gómez-Díaz *et al.* 2009 |
| *C. borealis* | 6109144 | Terreros Is.- Spain E coast | FJ755601 | Gómez-Díaz *et al.* 2009 |
| *C. borealis* | 6109148 | Terreros Is.- Spain E coast | FJ755561 | Gómez-Díaz *et al.* 2009 |
| *C. borealis* | L053523 | Berlengas- Portugal | FJ755602 | Gómez-Díaz *et al.* 2009 |
| *C. borealis* | L22517 | Berlengas- Portugal | FJ755571 | Gómez-Díaz *et al.* 2009 |
| *C. borealis* | L22525 | Berlengas- Portugal | FJ755552 | Gómez-Díaz *et al.* 2009 |
| *C. borealis* | L22538 | Berlengas- Portugal | FJ755603 | Gómez-Díaz *et al.* 2009 |
| *C. borealis* | L29028 | Berlengas- Portugal | FJ755604 | Gómez-Díaz *et al.* 2009 |
| *C. borealis* | L44532 | Berlengas- Portugal | FJ755601 | Gómez-Díaz *et al.* 2009 |
| *C. borealis* | L44600 | Berlengas- Portugal | FJ755605 | Gómez-Díaz *et al.* 2009 |
| *C. borealis* | L22501 | Berlengas- Portugal | FJ755606 | Gómez-Díaz *et al.* 2009 |
| *C. borealis* | L44497 | Berlengas- Portugal | FJ755607 | Gómez-Díaz *et al.* 2009 |
| *C. borealis* | L44842 | Berlengas- Portugal | FJ755606 | Gómez-Díaz *et al.* 2009 |
| *C. borealis* | L068402 | Madeira | FJ755552 | Gómez-Díaz *et al.* 2009 |
| *C. borealis* | 5401 | Madeira | FJ755570 | Gómez-Díaz *et al.* 2009 |
| *C. borealis* | 5402 | Madeira | FJ755608 | Gómez-Díaz *et al.* 2009 |
| *C. borealis* | 5404 | Madeira | FJ755609 | Gómez-Díaz *et al.* 2009 |
| *C. borealis* | 5405 | Madeira | FJ755610 | Gómez-Díaz *et al.* 2009 |
| *C. borealis* | 5411 | Madeira | FJ755557 | Gómez-Díaz *et al.* 2009 |
| *C. borealis* | 5421 | Madeira | FJ755611 | Gómez-Díaz *et al.* 2009 |
| *C. borealis* | 5424 | Madeira | FJ755612 | Gómez-Díaz *et al.* 2009 |
| *C. borealis* | 5431 | Madeira | FJ755557 | Gómez-Díaz *et al.* 2009 |

Table S2 (continued)

| **SPECIES** | **ID CODE** | **Geographic Locality** | **GenBank Ac.No** | **Dataset** |
| --- | --- | --- | --- | --- |
| *C. borealis* | L070625 | Madeira | FJ755552 | Gómez-Díaz *et al.* 2009 |
| *C. borealis* | 6002 | La Palma- Canary Is. | FJ755613 | Gómez-Díaz *et al.* 2009 |
| *C. borealis* | 6004 | La Palma- Canary Is. | FJ755614 | Gómez-Díaz *et al.* 2009 |
| *C. borealis* | 6010 | La Palma- Canary Is. | FJ755554 | Gómez-Díaz *et al.* 2009 |
| *C. borealis* | 6012 | La Palma- Canary Is. | FJ755563 | Gómez-Díaz *et al.* 2009 |
| *C. borealis* | 6016 | La Palma- Canary Is. | FJ755615 | Gómez-Díaz *et al.* 2009 |
| *C. borealis* | 6017 | La Palma- Canary Is. | FJ755615 | Gómez-Díaz *et al.* 2009 |
| *C. borealis* | 6018 | La Palma- Canary Is. | FJ755616 | Gómez-Díaz *et al.* 2009 |
| *C. borealis* | 6019 | La Palma- Canary Is. | FJ755615 | Gómez-Díaz *et al.* 2009 |
| *C. borealis* | 1212 | Montaña Clara- Lanzarote- Canary Is. | KC888883 | Extant, this study |
| *C. borealis* | 1213 | Montaña Clara- Lanzarote- Canary Is. | KC888884 | Extant, this study |
| *C. borealis* | 1214 | Montaña Clara- Lanzarote- Canary Is. | KC888885 | Extant, this study |
| *C. borealis* | 1215 | Montaña Clara- Lanzarote- Canary Is. | KC888886 | Extant, this study |
| *C. borealis* | 1217 | Montaña Clara- Lanzarote- Canary Is. | KC888888 | Extant, this study |
| *C. borealis* | 1220 | Montaña Clara- Lanzarote- Canary Is. | KC888889 | Extant, this study |
| *C. borealis* | 1225 | Montaña Clara- Lanzarote- Canary Is. | KC888891 | Extant, this study |
| *C. borealis* | 1226 | Montaña Clara- Lanzarote- Canary Is. | KC888892 | Extant, this study |
| *C. borealis* | 1227 | Montaña Clara- Lanzarote- Canary Is. | KC888893 | Extant, this study |
| *C. borealis* | 1229 | Montaña Clara- Lanzarote- Canary Is. | KC888894 | Extant, this study |
| *C. borealis* | 1238 | Montaña Clara- Lanzarote- Canary Is. | KC888897 | Extant, this study |
| *C. borealis* | 1239 | Montaña Clara- Lanzarote- Canary Is. | KC888898 | Extant, this study |
| *C. borealis* | 1240 | Montaña Clara- Lanzarote- Canary Is. | KC888899 | Extant, this study |
| *C. borealis* | 1242 | Montaña Clara- Lanzarote- Canary Is. | KC888900 | Extant, this study |
| *C. borealis* | 1243 | Montaña Clara- Lanzarote- Canary Is. | KC888901 | Extant, this study |
| *C. borealis* | 1244 | Montaña Clara- Lanzarote- Canary Is. | KC888902 | Extant, this study |
| *C. borealis* | 1245 | Montaña Clara- Lanzarote- Canary Is. | KC888903 | Extant, this study |
| *C. borealis* | 1246 | Montaña Clara- Lanzarote- Canary Is. | KC888883 | Extant, this study |
| *C. borealis* | 1247 | Montaña Clara- Lanzarote- Canary Is. | FJ755614 | Extant, this study |
| *C. borealis* | 1248 | Montaña Clara- Lanzarote- Canary Is. | KC888904 | Extant, this study |
| *C. borealis* | 6134583 | Montaña Clara- Lanzarote- Canary Is. | KC888879 | Extant, this study |
| *C. borealis* | 6134585 | Montaña Clara- Lanzarote- Canary Is. | FJ755552 | Extant, this study |
| *C. borealis* | 6134590 | Montaña Clara- Lanzarote- Canary Is. | FJ755586 | Extant, this study |
| *C. borealis* | 6134593 | Montaña Clara- Lanzarote- Canary Is. | KC888880 | Extant, this study |
| *C. borealis* | 6134596 | Montaña Clara- Lanzarote- Canary Is. | KC888903 | Extant, this study |
| *C. borealis* | 6134597 | Montaña Clara- Lanzarote- Canary Is. | KC888881 | Extant, this study |
| *C. borealis* | 6134600 | Montaña Clara- Lanzarote- Canary Is. | FJ755601 | Extant, this study |
| *C. borealis* | 6134604 | Montaña Clara- Lanzarote- Canary Is. | KC888882 | Extant, this study |
| *C. borealis* | 6134594 | Montaña Clara- Lanzarote- Canary Is. | KC888882 | Extant, this study |
| *C. borealis* | 1216 | Montaña Clara- Lanzarote- Canary Is. | KC888887 | Extant, this study |
| *C. borealis* | 1221 | Montaña Clara- Lanzarote- Canary Is. | FJ755552 | Extant, this study |
| *C. borealis* | 1222 | Montaña Clara- Lanzarote- Canary Is. | KC888890 | Extant, this study |
| *C. borealis* | 1223 | Montaña Clara- Lanzarote- Canary Is. | FJ755552 | Extant, this study |
| *C. borealis* | 1224 | Montaña Clara- Lanzarote- Canary Is. | KC888903 | Extant, this study |
| *C. borealis* | 1230 | Montaña Clara- Lanzarote- Canary Is. | KC888895 | Extant, this study |
| *C. borealis* | 1231 | Montaña Clara- Lanzarote- Canary Is. | KC888880 | Extant, this study |
| *C. borealis* | 1232 | Montaña Clara- Lanzarote- Canary Is. | KC888896 | Extant, this study |
| *C. borealis* | 1233 | Montaña Clara- Lanzarote- Canary Is. | KC888896 | Extant, this study |
| *C. borealis* | 1234 | Montaña Clara- Lanzarote- Canary Is. | KC888890 | Extant, this study |
| *C. borealis* | 1235 | Montaña Clara- Lanzarote- Canary Is. | FJ755552 | Extant, this study |
| *C. borealis* | 1236 | Montaña Clara- Lanzarote- Canary Is. | KC888903 | Extant, this study |
| *C. borealis* | 1241 | Montaña Clara- Lanzarote- Canary Is. | FJ755606 | Extant, this study |
| *C. borealis* | Alegr1Cons1 | Alegranza- Lanzarote- Canary Is. | KC888905 | Extinct, this study |
| *C. borealis* | Alegr1Cons2 | Alegranza- Lanzarote- Canary Is. | KC888906 | Extinct, this study |
| *C. borealis* | Alegr2Cons1 | Alegranza- Lanzarote- Canary Is. | KC888907 | Extinct, this study |
| *C. borealis* | Alegr2Cons2 | Alegranza- Lanzarote- Canary Is. | FJ755609 | Extinct, this study |
| *C. borealis* | Alegr3Cons1 | Alegranza- Lanzarote- Canary Is. | KC888908 | Extinct, this study |
| *C. borealis* | Alegr2Cons2 | Alegranza- Lanzarote- Canary Is. | FJ755555 | Extinct, this study |
| *C. borealis* | MC1Cons | Montaña Clara- Lanzarote- Canary Is. | FJ755565 | Extinct, this study |

Table S2 (continued)

| **SPECIES** | **ID CODE** | **Geographic Locality** | **GenBank Ac.No** | **Dataset** |
| --- | --- | --- | --- | --- |
| *C. borealis* | MC1.2Cons1 | Montaña Clara- Lanzarote- Canary Is. | KC888909 | Extinct, this study |
| *C. borealis* | MC1.2Cons2 | Montaña Clara- Lanzarote- Canary Is. | KC888910 | Extinct, this study |
| *C. borealis* | MC3.2Cons1 | Montaña Clara- Lanzarote- Canary Is. | FJ755583 | Extinct, this study |
| *C. borealis* | MC3.2Cons2 | Montaña Clara- Lanzarote- Canary Is. | KC888911 | Extinct, this study |
| *C. borealis* | MC4Cons1 | Montaña Clara- Lanzarote- Canary Is. | FJ755584 | Extinct, this study |
| *C. borealis* | MC4Cons2 | Montaña Clara- Lanzarote- Canary Is. | KC888912 | Extinct, this study |
| *C. borealis* | MC5Cons1 | Montaña Clara- Lanzarote- Canary Is. | KC888913 | Extinct, this study |
| *C. borealis* | MC5Cons2 | Montaña Clara- Lanzarote- Canary Is. | KC888914 | Extinct, this study |
| *C. borealis* | MC5.2Cons1 | Montaña Clara- Lanzarote- Canary Is. | KC888915 | Extinct, this study |
| *C. borealis* | MC5.2Cons2 | Montaña Clara- Lanzarote- Canary Is. | KC888916 | Extinct, this study |
| *C. borealis* | MC6Cons1 | Montaña Clara- Lanzarote- Canary Is. | KC888917 | Extinct, this study |
| *C. borealis* | MC6Cons2 | Montaña Clara- Lanzarote- Canary Is. | KC888918 | Extinct, this study |
| *C. borealis* | MC6.2Cons1 | Montaña Clara- Lanzarote- Canary Is. | KC888919 | Extinct, this study |
| *C. borealis* | MC6.2Cons2 | Montaña Clara- Lanzarote- Canary Is. | KC888920 | Extinct, this study |
| *C. borealis* | MC7Cons1 | Montaña Clara- Lanzarote- Canary Is. | KC888921 | Extinct, this study |
| *C. borealis* | MC7Cons2 | Montaña Clara- Lanzarote- Canary Is. | KC888922 | Extinct, this study |
| *C. borealis* | MC24Cons1 | Montaña Clara- Lanzarote- Canary Is. | KC888923 | Extinct, this study |
| *C. borealis* | MC24Cons2 | Montaña Clara- Lanzarote- Canary Is. | KC888924 | Extinct, this study |
