# Supplementary material for: Population connectivity buffers genetic diversity loss in a seabird
Source: Front Zool. 2013 May 20;10:28. doi: 10.1186/1742-9994-10-28 (PMC3662614; doi:10.1186/1742-9994-10-28)
Supplement: Additional file 5: Table S3 — Primers used for the amplification of a fragment corresponding to the mtDNA control region. [file 1742-9994-10-28-S5.docx]

**Table S3.** Primers used for amplification of a fragment corresponding to the mtDNA control region

| Primer Name | Sequence (5´-3´) | Product size |
| --- | --- | --- |
| CaloCRF1  CaloCRR1 | CAAACACATTCAATGCATG  TTTGTCCTGCTACGATTGA | 218 bp |
| CaloCRF1  CaloCRR2 | CAAACACATTCAATGCATG  GTTTCTGGTACTAGGGAC | 113 bp |
| CaloCRF2  CaloCRR1 | CCCTTAAGCCCAATAGTCC  TTTGTCCTGCTACGATTGA | 138 bp |
